# Identifying and subtyping dyscalculia in a sample of children with and without dyscalculia — a data-driven approach

**DOI:** 10.3389/fpsyg.2025.1590581

**Published:** 2025-09-23

**Authors:** Christian Kißler, Jörg-Tobias Kuhn

**Affiliations:** Methods in Empirical Educational Research, Rehabilitation Sciences, TU Dortmund University, Dortmund, Germany

**Keywords:** dyscalculia, subtypes, learning disorder, working memory, reading fluency, mathematical competence

## Abstract

**Introduction:**

Dyscalculia is a very heterogeneous disorder. This is illustrated by the fact that numerous possible subtypes have been described in previous studies. Therefore, the present study addresses the question of whether children with dyscalculia form a homogeneous group that can be distinguished from children without dyscalculia or whether distinct dyscalculia subtypes should be assumed.

**Methods:**

A sample of 1,015 children was analyzed in a data-driven subtyping approach (mixture model analysis). 93 of these children were identified as dyscalculic (criterion: percentage rank <10) with a standardized test (HRT 1–4) to examine how these children were distributed across the identified subtypes. Various cognitive performance domains that were measured with standardized tests were included in the analyses: mathematical skills (basic numerical processing, complex number processing, calculation), working memory, reading fluency, and intelligence. To check the subgrouping results for robustness, four different approaches were used, which differed with respect to which variables were included in the mixture model analysis (only mathematical skills: $n_{1}$ = 1,015/ all variables: $n_{2}$ = 478; $n_{2}$ with a reduced sample size according to missing data) and to what extent the measured results were aggregated into constructs (construct level) or considered as individual test results (subtest level).

**Results:**

In three of these four different subtyping approaches, at least one of the identified subgroups showed significant deficits in mathematical skills and included disproportionately many children with dyscalculia. Furthermore, one of these three approaches (the subtyping analysis at the subtest level based on mathematical skills only) suggests that there may be two subtypes of children with dyscalculia: a subtype with mild deficits and a severely impaired subtype. In one approach (subtyping analysis at the construct level with all variables included), children with dyscalculia were not identified as a separable group.

**Discussion:**

In summary, dyscalculia subtypes (as well as children with dyscalculia in general) do not seem to be clearly distinguishable from children without dyscalculia: the boundaries are fluid. For educational practice, this fluent transition between dyscalculic and non-dyscalculic children means that all children who have difficulties in mathematics should be supported and not only those who are classified as dyscalculic.

## 1 Introduction

Since this study focuses on subtyping children with dyscalculia (CwD), dyscalculia will first be described, followed by a discussion of subtyping approaches (subsection 1.1). Subsequently, the current state of research regarding the subtyping of CwD is presented (subsection 1.2).

### 1.1 Dyscalculia and subtyping approaches

Severe difficulties in the domain of mathematics are described using different terms, although they refer to similar or identical phenomena. The DSM-5-TR (American Psychiatric Association, 2022) lists *specific learning disorders* that can be coded with the specifier *with impairment in mathematics*, while the ICD-11 (World Health Organization, 2020) refers to *developmental learning disorder with impairment in mathematics (6A03.2)*. In the DSM-5-TR, dyscalculia is mentioned as an alternative term for a pattern of difficulties in mathematics. The following section provides a more detailed description of dyscalculia in the context of this study.

Dyscalculia is understood as a developmental learning disorder which is characterized by a lack of “skills related to mathematics or arithmetic, such as number sense, memorization of number facts, accurate calculation, fluent calculation, and accurate mathematic reasoning” (ICD-11: World Health Organization, 2020), insofar as the deficits (a) cannot be explained by another disorder (e.g., an intellectual impairment) and (b) occur even though the individual had access to education (World Health Organization, 2020). The DSM-5-TR describes this disorder very similar (American Psychiatric Association, 2022). Both the ICD-11 and DSM-5-TR list dyscalculia as a neurodevelopmental disorder (World Health Organization, 2020; American Psychiatric Association, 2022). Overall, there are different approaches that aim to explain children’s difficulties in arithmetic and mathematical reasoning (e.g., Butterworth, 2005; Noël and Rousselle, 2011).

Mathematical skills encompass complex subdomains, some of which appear to stand alone and thus form isolated constructs: in fact, different areas of mathematical abilities can be impaired in CwD, which implies that needs of CwD can vary substantially (Skagerlund and Träff, 2016; Haberstroh and Schulte-Körne, 2019). Therefore, evidence-based formation of different subareas of mathematical competencies makes sense from a theoretical and practical perspective.

Different structural models of mathematical skills and competencies have been suggested. According to factor analytic results of Kuhn et al. (2017), mathematical skills can be categorized into three subdomains: basic numerical processing (BNP), complex number processing (CNP) and calculation competencies. BNP is also known as core number competencies and is characterized by straightforward tasks, such as dot counting and the comparison of magnitudes (Reeve et al., 2012; Kuhn et al., 2017). CNP encompasses more complex mathematical precursor skills such as (a) locating a number on a number line or (b) transcoding/transforming auditorily presented numbers into written Arabic symbols, for example (Nuerk et al., 2006; Kuhn et al., 2017). Calculation implies performing concrete arithmetic operations such as addition, subtraction, and multiplication.

Because there are distinct mathematical abilities, dyscalculia may also affect these to different degrees; consequently, several theories have been proposed to explain challenges of CwD. Some approaches link dyscalculia to theories of number processing: e.g., to the *Approximate Number System* (ANS). This is thought to handle quantities larger than four in an approximate manner, and complements the *Object Tracking System* (OTS), which is assumed to register quantities up to four exactly and instantaneously (Piazza et al., 2010; Lamb et al., 2024).

Therefore, one perspective suggests that difficulties in mathematics derive from an impaired ANS (Feigenson et al., 2004; Noël and Rousselle, 2011; Lamb et al., 2024). Indeed, evidence shows that 10-year-old CwD perform at the level of 5-year-old typically developing children when estimating dot quantities (Piazza et al., 2010). In Accordance with the ANS hypothesis, CwD should show deficits in the following tasks, for example: in pure number-comparison tasks, mixed comparison tasks of quantities and numbers, and dot enumerations that go beyond four (Lamb et al., 2024). But they should not show such deficits in dot enumeration tasks from one to three (Lamb et al., 2024).

Another perspective to explain deficits in mathematics suggests a deficit in the ability to access quantity representations from symbols: the *Access Deficit Hypothesis* (Rousselle and Noël, 2007; Noël and Rousselle, 2011; Skagerlund and Träff, 2016). If this hypothesis is correct, CwD should show deficits in specific task domains: for example, in pure number-comparison tasks as well as in mixed comparisons of quantities and numbers (Lamb et al., 2024). However, they should not exhibit deficits when counting dots – regardless of the number of dots (Lamb et al., 2024).

Thus, it becomes evident that different explanatory approaches for difficulties observed in CwD should correspond to distinct patterns of deficits. Nevertheless, these approaches do not need to be exclusive: there could be separate subtypes of CwD whose difficulties stem from different underlying causes.

In addition to different deficient math skills, other domain general abilities such as working memory, and attention or reading performance are often discussed in the context of dyscalculia, as many CwD appear to have pronounced deficits in these areas (e.g., Schuchardt et al., 2008; Mähler and Schuchardt, 2011; Haberstroh and Schulte-Körne, 2019; Kißler et al., 2020; Kißler et al., 2021). Whether such deficits really apply to all CwD, or whether these concern (in particular) specific subtypes, will be discussed in the following sections in more detail.

Notably, not only CwD, but also children with other learning disorders – e.g., reading disorder – exhibit difficulties in domain-general abilities (e.g., Menghini et al., 2011) and even comorbidities of different learning disorders are common (e.g., Gross-Tsur et al., 1996). This raises the question of whether different learning disorders are truly separable or whether they are more closely related. It may be worth considering that disorders such as dyslexia and dyscalculia should not be viewed categorically, but rather within a dimensional framework (Peters and Ansari, 2019).

If dyslexia and dyscalculia were distinct, their deficits would be more likely to be additive (Kißler et al., 2020), as different underlying causes would then be present and co-occur in children with both disorders. Indeed, Kißler et al. (2020) found evidence for the additivity of cognitive deficit profiles in children with arithmetic and reading difficulties. Nevertheless, the high comorbidity between reading and arithmetic disorders requires further investigation. Interestingly, CwD showed contradictory results in working memory tasks assessing visuospatial working memory: an effect was found in matrix span tasks, whereas no such effect was found in Corsi block tasks (Kißler et al., 2020). These results contradict Schuchardt and Mähler (2010), who found such problems in Cosi block tasks, whereas Landerl et al. (2009), in turn, did not find such deficits. In a meta-analysis (Viesel-Nordmeyer et al., 2023) of 74 studies additivity for deficits in math and reading skills was found, whereas underadditivity was found in executive functions (inhibition, shifting, and updating). However, for example, de Weerdt et al. (2013) found no interaction effect for inhibition and concluded that children with reading disabilities show inhibition deficits related to alphanumeric stimuli, but children with mathematical disabilities do not.

Contradicting results may point to the heterogeneity of children with learning disorders and especially of CwD. But if results vary across studies (as shown above), it raises the question of whether the same disorders are investigated or whether different subtypes are studied, for example due to the use of different diagnostic instruments. For this reason, the identification of subtypes is an important topic of research.

In principle, there are two approaches to state subtypes of disorders such as dyscalculia: top-down and bottom-up (Salvador et al., 2019; Kißler et al., 2021). In top-down approaches, individuals are grouped (=subtyped) based on (a) observations that tend to be unsystematic or (b) theories that are more the result of logical reasoning than of systematic approaches that are evidence-based: for example, children who have difficulties with arithmetic can be distinguished from children who have difficulties with arithmetic and reading *a priori* (Rourke et al., 1971; Rourke and Finlayson, 1978; Ozols and Rourke, 1988; Rourke, 1993). However, it is unclear whether each of these two groups includes children with homogeneous cognitive profiles. Of course, these a priori distinguished groups can be compared with each other with respect to their performance in specific sub-areas, and in some cases, differences will undoubtedly be found. But this does not necessarily clarify the question of whether specific subtypes of a disorder were studied, or whether the mere effect of a comorbidity was analyzed.

Nevertheless, it should be noted that top-down approaches are justified and, in some contexts (for example in educational or clinical settings in practice), could be the only realistically applicable approach. Furthermore, the top-down approach can be used to identify entirely new disorders, if unsystematic observations lead to the conclusion that specific (behavioral of cognitive) patterns cannot be explained by previously described disorders. This is like when Kanner (1943) and Asperger (1944) categorized children (belonging to the described group vs. not belonging to the described group), based on observations, and described specific characteristics of the groups they found. From that point on, children with similar characteristics were assigned top-down to those categories, but these categories were later merged into the autism spectrum disorder because of new evidence (e.g., Lord et al., 2012; World Health Organization, 2020; Habermann and Kißler, 2022).

The bottom-up approach is opposed to the top-down approach. In the bottom-up approach, data on children’s performance in different subdomains is collected, and then these children are divided into subgroups in a data-driven way (i.e., evidence-based and systematic, by applying specific statistical methods or algorithms). Next, these subgroups are compared with each other (e.g., Bartelet et al., 2014; Kißler et al., 2021). Top-down and bottom-up approaches do not always lead to the same groupings: Thus, in a data-driven research approach, Kißler et al. (2021) were unable to find a subgroup of children with dyscalculia (CwD) that stood out in terms of their reading competencies.

Several studies (as described below) have already attempted to describe subgroups of dyscalculia top-down or bottom-up. In some cases, very heterogeneous research results were observed. However, if the bottom-up approach is taken seriously, it would need to be applied not only on children with a specific disorder (e.g., dyscalculia) to identify subgroups of CwD, but also to a large sample of both impaired and unimpaired children. This would allow for an analysis of (1) whether dyscalculia can be identified as a homogeneous disorder or (2) whether children with specific subtypes of dyscalculia can be distinguished from each other and from children without dyscalculia. The goal of this study is to investigate precisely that. If specific subtypes of dyscalculia do indeed exist, this might imply that these subtypes, although having similarities, represent distinct disorders that could have different causes and might require different interventions.

### 1.2 State of research on the subtyping of dyscalculia

As described above, there are two contrasting approaches in research on subtyping (Kißler et al., 2021): top-down and bottom-up. Presumably, different findings on subtypes of CwD can partly be explained by different methodological approaches, small sample sizes that were analyzed, and the consideration of just a few cognitive sub-performance domains in the formation of subtypes, as well as by how dyscalculia was defined.

Rourke and the research team around him were among the first in researching subtypes of CwD (e.g., Ozols and Rourke, 1988; Rourke, 1993). They categorized CwD into three groups: (1) children who struggled with arithmetic, reading, and spelling, (2) children who had poor reading and spelling abilities but showed relatively better skills in arithmetic (although still deficient), and (3) children who had average or above-average reading and spelling abilities but experienced mathematical difficulties. The qualitative nature of arithmetic errors differed among these groups (Rourke, 1993). For example, group 3 had issues with accurate calculation due to poor handwriting, misread mathematical symbols, performed arithmetic operations incorrectly, and showed difficulties in accessing the required calculation rules from long-term memory (Rourke, 1993). In contrast, children in group 2 made mistakes that could be linked to their reading problems (Rourke, 1993). Consequently, this research provides evidence that reading skills are linked to specific problem areas in some CwD and that reading skills have to be considered when discussing dyscalculia subtypes.

Accessing information from long term memory is a common problem of a subtype in CwD that was also described by Skagerlund and Träff (2016). These authors described two subtypes of CwD, namely the General Dyscalculia Subtype (GDS) and the Arithmetic Fact Dyscalculia Subtype (AFDS). The GDS had deficits in the innate ANS: this means that in children with GDS, the ANS – which is responsible for representing numerosities – showed an arrangement of numbers on a mental number line that was too imprecise for their chronological age (Halberda and Feigenson, 2008; Skagerlund and Träff, 2016). In contrast, the AFDS was characterized by another deficit: accessing magnitude information from symbols was impaired (access deficit hypothesis; Rousselle and Noël, 2007; Skagerlund and Träff, 2016). Moreover, the AFDS did not show deficits in non-symbolic processing, whereas children of the GDS showed such deficits (Skagerlund and Träff, 2016).

In a research project with 226 children (3rd to 6th grade) with math learning difficulties (percentile rank, abbreviated as PR, of < 16 in an arithmetic fluency test), Bartelet et al. (2014) used a data-driven approach to identify subtypes of children with math difficulties by focusing on different variables: Arabic number knowledge, counting, number line estimation, approximate number knowledge (e.g., dot comparison task), spatial short-term working memory, verbal short-term working memory, and intelligence. Bartelet et al. (2014) found six different dyscalculia subtypes, each with distinct cognitive characteristics: (1) The *weak mental number line subtype*, which exhibited low performance in number line tasks but demonstrated strong skills in approximate numerical knowledge and Arabic numeral knowledge; (2) The *weak ANS subtype*, characterized by difficulties in approximate number knowledge and number line tasks, but with a high IQ and a good performance in spatial short-term working memory compared to other subtypes. This subtype shared similarities with the GDS subtype described by Skagerlund and Träff (2016); (3) The *spatial difficulties subtype*, which struggled primarily with spatial short-term working memory and approximate numerical knowledge. Additionally, this subtype seemed to have difficulties in verbal short-term working memory and in solving number line tasks; (4) The *access deficit subtype*: In this subtype, difficulties in counting and Arabic numerical knowledge were found; (5) The *no numerical cognitive deficit subtype*, which showed no impairments in any area and very high verbal short-term working memory; (6) The *garden* var*iety subtype*, characterized by multiple smaller deficits across various areas. This subtype performed well in number line tasks but had a lower IQ.

Bartelet et al. (2014) found subtypes in children with math learning difficulties that were characterized by varying abilities/ problems in different mathematical areas and other cognitive domains. Rourke (1993) also found that mathematical deficits differ qualitatively between children with math difficulties (Rourke, 1993). The research of Kißler et al. (2021) even suggests that mathematical skills may be the most relevant factors in subtyping CwD: This means that children who meet the common criterion for dyscalculia vary in terms of their mathematical abilities in such a way, that specific subgroups can also be found among dyscalculic children with regard to their arithmetic abilities.

Kißler et al. (2021) analyzed two samples (one included 71 CwD, the other 103 CwD) using mixture model analyses to identify subgroups of CwD based on a broad range of constructs (attention, intelligence, reading fluency, working memory, and different mathematical skills). They found two subgroups that differed in particular with respect to their mathematical performance and their attention: the so-called *subtype 2* was inferior to *subtype 1* in terms of performance in both subareas. Overall, subtype 2 seemed to be more impaired than subtype 1. Intelligence, working memory, and reading fluency were not suitable for systematically distinguishing the two identified subtypes. The results were robust regardless of whether the analyses were conducted at the construct level or subtest level (e.g., construct level: *working memory*; subtest level for working memory: *matrix span* and *verbal span*) and whether only complete data sets or data sets with imputations to deal with missing data were used (Kißler et al., 2021).

The finding that CwD differ particularly in their mathematical abilities also fits to the approach of a recent research (Pedemonte et al., 2022). Starting with the assumption that CwD show difficulties in different mathematical subareas, the research team developed the UCSF Dyscalculia Subtyping Battery (DSB) with the aim to identify difficulties in specific mathematical subareas and different dyscalculia subtypes corresponding to these specific mathematical subareas top-down (arithmetic facts retrieval, arithmetical procedures, geometrical abilities and number processing). Thus, the subtypes were formed *a priori*, based on a specific conceptualization of dyscalculia. This test battery has been evaluated on a small sample of 93 children/ adolescents aged 7–16 years. 50 of them were diagnosed with dyslexia, 7 with ADHD, and 18 with dyslexia and ADHD. 18 children were typically developing. Although this study has considerable methodological limitations (e.g., very small sample size, wide age range from 7 to 16 years old, limited selection of statistical methods), the approach of subtyping CwD based on their deficits in different mathematical subareas using a test battery seems to be an interesting and consistent approach in view of the evidence from other studies (e.g., Skagerlund and Träff, 2016; Kißler et al., 2021).

The studies previously presented analyzed CwD using a top-down or bottom-up approach to examine the existence of subtypes of CwD. But these subtypes were either derived from preexisting theoretical assumptions about dyscalculia or – in cases where a bottom-up approach was used – affected children (with dyscalculia) were initially distinguished from unaffected children a priori: Thus, a top-down or an incomplete bottom-up approach was used, as a distinction (affected vs. unaffected children) was made top-down before subtyping bottom-up. However, the studies presented below analyzed samples that encompass both CwD and children without dyscalculia by using a bottom-up approach consistently.

Pieters et al. (2015) used model-based clustering-analyses to identify subgroups in a sample that encompassed 73 children with mathematical learning disabilities, 102 children with developmental coordination disorder, 99 children with both disorders, and 136 children without any of these disorders. Different approaches to cluster these children were performed: Thus, in one approach only mathematical variables were considered and, in another approach, mathematical and motor skills were considered to perform the cluster-analyses. In the first cluster approach (mathematical variables only), two clinically relevant clusters were found: one cluster showing deficits in number fact retrieval and procedural calculation, and the other cluster showing deficits in procedural calculation. In the other cluster approach (motor and mathematical skills), two clinically relevant clusters were identified as well: here, a subtype with deficits in number fact retrieval was found, too. In addition, this cluster also showed further difficulties (deficits in procedural calculation as well as below-average motor and visual-motor integration skills). The second cluster found within this approach showed deficits in procedural calculation and in addition visual-motor problems. Thus, both approaches (mathematical variables only vs. motor and mathematical skills) that were reported here produce similar results, which may indicate robustness of the results.

Salvador et al. (2019) used a data-driven/ bottom-up approach in a sample of 192 children (age: 8–11 years) and they found 4 clusters. They used a hierarchical cluster analysis (Ward method with squared Euclidean distance) and focused on a small range of cognitive domains: phonological and visuospatial working memory, visuospatial and visuoconstructional processing, and symbolic as well as nonsymbolic magnitude accuracy. Two of the clusters that were found exhibited difficulties typical for children with numeracy difficulties: cluster 1 showed low visuospatial abilities and the highest percentage frequency of individuals with identified math difficulties; cluster 2 showed low magnitude processing accuracy. Both clusters showed average or increased intelligence. The other two clusters showed average (cluster 3) or high performance (cluster 4) in some areas. Limitations of the study are the small sample size and the focus on only a few (mathematical) performance domains.

Like Pieters et al. (2015) and Salvador et al. (2019), Huijsmans et al. (2020) also did not only focus on CwD: their aim was to discern distinct cognitive profiles among a group of 281 fourth-grade children by assessing their skills in fundamental arithmetic and more advanced mathematical abilities. Only one of four identified cognitive profiles showed significant mathematical deficits (=the low-achieving profile). However, 33% of the children in this sample (94 out of 281) could be assigned to this low-achieving profile, so it does not seem to be a profile that explicitly includes CwD, because the prevalence for dyscalculia is considerably lower. In summary, Huijsmans et al. (2020) did not succeed in distinguishing children with a low-achieving profile in mathematics from children who met the diagnostic criteria for dyscalculia. Possibly, this problem can be explained with the small sample size: Therefore, systematic differences between CwD and children with a low-achieving profile may not be systematically identified due to a lack of data/ power.

Overall, it is to be noted that not all subtyping studies were able to distinguish CwD from children without dyscalculia. Regardless of whether (1) only children with dyscalculia or (2) children with and without dyscalculia were investigated, some subtyping approaches lead to very different results, with other findings in turn (partially) coinciding. It is necessary to conduct a subtyping analysis with a view to numerous cognitive sub-performance areas and based on a large sample to generate further and valid findings in this research area. Furthermore, such a subtyping analysis with another approach can be used to check whether the findings from previous studies can be affirmed and/ or reproduced.

### 1.3 Research question and aim of this study

In this study, a large sample of children with and without dyscalculia is used in a bottom-up (i.e., data-driven) approach to address the following question: To what extent do children with dyscalculia form a homogeneous group that can be distinguished from children without dyscalculia? This question includes both, (1) the interest in examining whether CwD can be distinguished from children without dyscalculia, and (2) the question of whether such a group of CwD forms one homogeneous group, or whether distinct dyscalculia subtypes should be assumed. Because the body of research on the existence of dyscalculia subtypes is ambiguous, a quantitative-exploratory approach is taken to pursue this research endeavor. As this is an exploratory, quantitative data analysis with open outcomes – including whether any subtypes can be identified at all and whether CwD can be distinguished from children without dyscalculia by using a data-driven approach – no research hypotheses are stated. Instead, the research question is answered by using standardized, statistical methods, and further analyses are conducted to interpret the results. The data analysis approach is presented in subsection 2.3.

## 2 Methods

### 2.1 Sample

The analyzed sample included a total of 1,015 children from elementary schools in Germany and was part of a large-scale investigation of mathematical skills. 530 of these children were female, 483 of them were male, and the gender of 2 children was not recorded. All children were in the 2nd to 4th grade at the time of the survey (grade 2: 333 children; grade 3: 422 children; grade 4: 260 children). Therefore, all children were at the age to attend elementary school: the mean age was 8.98 years (SD = 0.87), although the exact age was not recorded for 565 children. Parental consent was obtained prior to testing.

### 2.2 Tests

#### 2.2.1 Diagnostic test for assessing dyscalculia

The HRT 1–4 (“Heidelberger Rechentest 1–4”) is a pen-and-paper speed test designed to assess basic mathematical knowledge/ competencies and is composed of two scales: *arithmetic operations* and *numerical-logical and visual–spatial skills* (Haffner et al., 2005). These two scales were combined to produce a total score. The *arithmetic operations* scale includes six subtests, which are addition, subtraction, multiplication, division, fill-the-gap tasks, and greater/less comparisons; the scale has a retest reliability of 0.93 (Haffner et al., 2005). The *numerical-logical and visual–spatial skills* scale includes five subtests, which are numerical series, length estimation, counting cubes, counting magnitudes, and connecting numbers; this scale has a retest reliability of 0.87 (Haffner et al., 2005). Thus, the two scales assess different abilities that are related to mathematical competencies. According to the S3-guideline, the HRT 1–4 is considered a suitable instrument for the diagnosis of mathematical learning disorders (Arbeitsgemeinschaft der Wissenschaftlichen Medizinischen Fachgesellschaften, 2018).

The overall score of this test was used to decide whether the children in the study met the criterion for dyscalculia and to examine how children identified as dyscalculic were distributed across the identified subtypes. Thus, the results of the HRT 1–4 were used to analyze whether there are specific subgroups that include only (or predominantly) CwD. T-score norms (which have an overall mean of 50 and a standard deviation of 10) are available for each quarter of a school year. The test was administered in a group setting, either at the Department of Psychology at the University of Münster or in a classroom.

#### 2.2.2 Intelligence

To assess intelligence, two tests were used: CFT 1-R or CFT 20-R. These are language free group tests. Because the data come from a study that focused on children with learning disorders (reading disorders as well) and include children with a mother tongue other than German, a non-verbal intelligence test was used. CFT 1-R was used to test the intelligence of children in grades 2 and 3 (retest-reliability: 0.95; Weiß and Osterland, 2013). The CFT 20-R was used to test the intelligence of children in grade 4 (the retest-reliability reaches from 0.80 to 0.82 and the consistence coefficient is 0.95; Weiß, 2006).

CFT 1-R consists of two parts: Part 1 (perception-based performance) encompasses substitution tasks, mazes and similarity tasks; part 2 (figural reasoning) comprises classification tasks as well as matrices and children have to complete sequences (Weiß and Osterland, 2013).

The CFT-20R, by contrast, consists of two structurally identical test parts, each containing four subtests: completing Sequences, classifications, matrices, and topological conclusions (Weiß, 2006). Compared to part 1, in part 2 the difficulty is increased (Weiß, 2006).

#### 2.2.3 Reading fluency

The Salzburger Lese-Screening (SLS 1–4) was used to assess reading fluency (Mayringer and Wimmer, 2003): the test with a parallel test reliability of at least 0.90 involves children reading a set of simple and unambiguous sentences (such as “Bananas are pink,” but in German). The children had to read and understand as many sentences as possible within a 3-min timeframe. To prove that the sentence was understood correctly, after reading each sentence, the children had to indicate whether the sentence was correct or incorrect by ticking a box. A child’s reading fluency was determined based on the number of correct responses they provide within the timeframe. This assessment of reading fluency requires a certain level of reading comprehension and basic knowledge of everyday facts.

#### 2.2.4 Working memory

To assess the visual–spatial working memory, the matrix span task (retest reliability: 0.61) of the CODY-M 2–4 was used (Kuhn et al., 2017). During this test, first a pattern of dots had to be memorized, then a distracting task was to be solved and after that, this dot pattern had to be remembered and reproduced correctly (Raddatz et al., 2017).

#### 2.2.5 Mathematical abilities

The CODY-M 2–4 battery (Kuhn et al., 2017) was used to measure different mathematical abilities. In the following, the descriptions of the mathematical tests are based on Raddatz et al. (2017) and Kißler et al. (2021). These tests belong to three constructs, which are described in the introduction and are the result of a factor analysis (Kuhn et al., 2017). According to the S3-guideline, the CODY-M 2-4 is considered a suitable instrument for the diagnosis of mathematical learning disorders (Arbeitsgemeinschaft der Wissenschaftlichen Medizinischen Fachgesellschaften, 2018).

##### 2.2.5.1 Basic numerical processing (BNP)

The construct BNP (retest reliability: 0.72) encompasses 3 subtests (Kuhn et al., 2017). With dot enumeration (counting 1–9 black dots as quickly and correctly as possible) the efficiency in counting was tested. Inspired by Defever et al. (2013), symbolic magnitude comparison tasks (two different Arabic numerals) and mixed magnitude comparison tasks (dots on the one side and an Arabic numeral on the other side) were used. Here, children had to decide, which entity was larger. For these three tests, an efficiency measure (median of correct response times/ number of correct responses) was used to assess the children’s performance in these tasks.

##### 2.2.5.2 Complex number processing (CNP)

Three subtests (number line, number sets, transcoding) of the construct CNP from the CODY-M 2–4 (Kuhn et al., 2017) were used in this study (retest reliability: 0.76; Kuhn et al., 2017). These at first sight very different tasks share the similarity of evaluating mathematical precursor skills that involve more advanced number processing (Nuerk et al., 2006; Kuhn et al., 2017). The *transcoding* tasks assessed the individuals’ ability to translate spoken numbers (presented through headphones) into written Arabic numerals. The task type *number sets*, based on Geary et al. (2009), was used to evaluate the individual’s efficiency in number processing across different presentation formats. In this speed test, an Arabic numeral (referred to as the target number) was displayed at the top of the screen, while numbers and/ or geometric figures (referred to as a number set) were shown at the bottom. Children were required to compare the sum of the elements represented by the number set at the bottom of the screen with the numeral above (either 5 or 9) and determine whether the sum matched the displayed number. The following example illustrates this type of task: If three geometric figures and the numeral 1 were displayed at the bottom as a number set and a 5 (in Arabic numeral form) was shown above as the target number, then the child would calculate 3 (geometric figures) + 1 (Arabic numeral) = 4, and had to compare the result (in this case: 4) with the target number shown above (in this case: 5) to determine if they are equal or unequal. Based on Siegler and Booth (2004), another task tested the accuracy of the *mental number line*: a number was displayed on the screen, and the children were required to use a computer mouse to place that number on an unscaled number line where only the endpoints were marked with 0 and 100.

##### 2.2.5.3 Calculation

The retest reliability of this subscale is 0.85 (Kuhn et al., 2017). The construct *Calculation* comprises the subtests (1) *addition*, (2) *subtraction* and (3) *multiplication*. The *addition* tasks involve simple arithmetic fact retrieval (e.g., to solve the task 1 + 6) and more difficult tasks (e.g., 183 + 18). The *subtraction* tasks are structured congruently to the addition tasks, but here numbers are not added but subtracted. The task category *multiplication* involves multiplication tasks to be solved by mental calculation (e.g., 6 * 17). All these tasks, which belong to the construct Calculation, focus on calculating with concrete numbers.

### 2.3 Statistical analysis

For all calculations and analyses version 4.3.3 of the statistical software R was used (R Core Team, 2024). The values/ scores of all variables underwent *T*-standardization, which produced *T*-scores. This led to a standardization of the sample’s data with a mean of 50 and a standard deviation of 10.

#### 2.3.1 General approach to identify possible subtypes of CwD

Because the aim of this study is to identify subtypes of dyscalculia, model-based clustering (parameterized finite Gaussian mixture models) based on the R-package *mclust* (Scrucca et al., 2016; Fraley et al., 2020; Fraley et al., 2024) was performed. In principal, the subtyping procedure was based on Kißler et al. (2021), although in contrast to the study of Kißler et al. (2021) the sample of this study was much larger, encompassed children with and without dyscalculia and the research question was not identical, too. However, the subtyping procedure was suitable because in this study, similar to the study by Kißler et al. (2021), the identification of clusters was intended in order to identify specific cognitive profiles or subgroups/ subtypes of children.

Model-based clustering was used to assess individuals’ cognitive profiles, with the Bayesian Information Criterion (BIC) determining the number of clusters (i.e., subgroups/ subtypes): Each participant in the sample was assigned to a distinct cluster based on the probability of belonging to one of the identified clusters (Vanbinst et al., 2015; Bouveyron et al., 2019). Clusters can vary in their geometric characteristics as their spatial orientation or their volume (equal vs. varying volume) and when determining the number of clusters, different models with those varying geometric characteristics were used to find out which combination of (1) number of clusters and (2) geometric characteristics of these models (= number-characteristics-combination) fit the data best (Makhabel et al., 2017; Bouveyron et al., 2019; Fraley et al., 2020). In the package *mclust*, each of these different models has a unique identifier that can be used to look up its geometric characteristics in the manual: for example, the identifier EEI stands for a model with diagonal clusters, equal volume, and equal shape (Fraley et al., 2020). The number-characteristics-combination with the lowest absolute BIC fits the data best (Vanbinst et al., 2015; Bouveyron et al., 2019). As the number-characteristics-combination with the lowest absolute BIC is the best trade-off to fit the data, the combination with the lowest absolute BIC was selected for further analyses (Pieters et al., 2015; Kißler et al., 2021).

#### 2.3.2 Dealing with missing values and variable levels

First, subtyping was performed by using the test results that were measured on subtest level (the subtests were described in subsection 2.2). In a second step, the same analyses were conducted on the higher-level constructs. The difference between subtest level and construct level is illustrated by the following example: The subtests *dot enumeration*, *symbolic magnitude comparison,* and *mixed magnitude comparison* were aggregated to the construct *Basic Numerical Processing (BNP)* by computing the mean of the three subtests. For both CNP and Calculation, the mean of the subtests belonging to the respective construct (see subsection 2.2.5) was calculated too. The construct approach reduces the impact of specific subtests on the subtyping outcome, as a single subtest might have a disproportionately strong differentiating effect.

Furthermore, for each of the two approaches described above (*subtest level* and *construct level*), another two-step approach was necessary to address the potential impact and distortion resulting from missing data. One of these two further steps was that the subtest-approach and the construct-approach were performed by only considering variables of various mathematical competencies measured using the *CODY-M 2–4 battery* (Kuhn et al., 2017), because only for those variables complete data sets were available ( $n_{1}$ = 1,015). This means, the following variables were not included in these two subtypings: *matrix span*, *intelligence* and *reading fluency*. Using mathematical variables only as one among other approaches for subtyping children is also in line with similar studies (e.g., Pieters et al., 2015).

Besides these analyses on subtest and construct level by only considering variables of different mathematical competencies measured by using the *CODY-M 2–4 battery* (Kuhn et al., 2017), the analyses were performed again for all variables (now also including *matrix span*, *intelligence* and *reading fluency*). But data sets with missing data had to be excluded from these analyses because the chosen statistical procedure can only be performed with complete data sets. Therefore, the sample size was reduced accordingly in this approach ( $n_{2}$ = 478). In contrast to Kißler et al. (2021), working with imputations was not purposeful here to deal with the missing values, because values were missing for too many subjects to obtain interpretable results after performing the imputation procedures: regarding intelligence 526 of 1,015 cases (=51.82%) were missing and regarding reading fluency 537 of 1,015 cases (=52.91%) were missing.

In summary, the total of four subtyping approaches was used to check systematically whether the results are robust: (1) subtest-approach by considering all variables (2) subtest-approach by only considering variables encompassing mathematical competencies, (3) construct-approach by considering all variables (4) construct-approach by only considering variables encompassing mathematical competencies.

#### 2.3.3 Methods to investigate the identified subtypes

The identified subgroups were compared with each other for differences and similarities. *Bayesian t-tests* and *post-hoc Tukey tests* as well as *frequentist t-tests* were used for this purpose. Unlike frequentist statistics, Bayesian methods, such as Bayesian *t*-tests, can not only be used to check if there is evidence for a difference between groups but also to inspect whether there is evidence for equality among the analyzed groups (Rouder et al., 2012; Wagenmakers et al., 2018). Bayesian analyses in this study were performed by using the R-package *BayesFactor* (Morey et al., 2024). A notable distinction between frequentist and Bayesian statistics is that Bayesian statistics do not yield *p*-values (e.g., *p* smaller than 0.05 means that there is evidence for the alternative hypothesis); instead, they provide Bayes Factors (BF). A BF below (1) 0.33 indicates moderate evidence supporting the null hypothesis, (2) 0.10 suggests strong evidence supporting the null hypothesis, (3) 0.033 indicates very strong evidence supporting the null hypothesis (Wagenmakers et al., 2018; Kißler et al., 2021). Conversely, a BF above (4) 3 suggests moderate evidence for the alternative hypothesis, (5) 10 suggests strong evidence for the alternative hypothesis, and (6) 30 suggests very strong evidence for the alternative hypothesis (Wagenmakers et al., 2018; Kißler et al., 2021). This means that results between 0.33 and 3 provide only indications of a trend, but the evidence is ambiguous. The results of the frequentist approach and results of the Bayesian analyses can lead to different conclusions, but if the results point into the same direction, this is a hint for robustness (Lindley, 1957; Sprenger, 2013; Wagenmakers et al., 2018).

*Cohen’s d* was used as an effect size to quantify the difference between subgroups and was computed with the R-package *lsr* (Navarro, 2015). Regardless of which subtest results were used for clustering, the identified subgroups were compared with respect to all subtests presented in the chapter about tests (except for the HRT 1–4, which was only used for identifying dyscalculia). The resulting cognitive profiles of the identified subgroups were visualized for all subtests, too.

$\chi^{2}$ -tests were used to check whether the children with dyscalculia (categorical variable: yes/ no) were evenly distributed across the subgroups and Cramér’s *V* was used to measure the effect size. Here, dyscalculia was defined by a percentage rank (PR) of less than 16, 10, or 5 in the diagnostic test for assessing dyscalculia (HRT 1–4: Haffner et al., 2005). For each of these PRs the *χ*^2^-test was performed, and Cramér’s *V* was calculated, too. Fisher’s exact test for count data was conducted to check the results of $\chi^{2}$ -tests for robustness. These analyses allow for examining the extent to which a different cut-off (PR) impacts the interpretation of the results.

Furthermore, it was necessary to investigate whether the subgroups show cognitive profiles that differ equally in all cognitive domains or whether the identified subgroups exhibit greater differences in particular cognitive domains than in other: If two subgroups run parallel to each other, this would mean that the more severely impaired subgroup of these two groups is equally inferior to the other subgroup in all subareas. If the cognitive profiles do not run parallel to each other, the more impaired subgroup shows more difficulties in specific cognitive subareas than in other cognitive subdomains. Parallelism was analyzed using profile analysis by using the R package *profileR* (Bulut and Desjardins, 2020; Bulut and Desjardins, 2022).

Parallelism was tested in two ways if more than two subgroups were identified: In a first step, all resulting subgroups were tested for parallelism in a joint analysis. If the result of this analysis becomes significant, at least some identified subgroups do not run parallel to each other. However, some subgroups might still run parallel to each other, while others do not. Therefore, in a second step, each subgroup was tested against each other subgroup to analyze if there is evidence for parallelism.

## 3 Results

In this section, the results of the different clustering approaches are presented. Each subsection focuses on the outcomes obtained when a specific clustering approach – indicated in the corresponding heading – was used.

**Figure 1:**
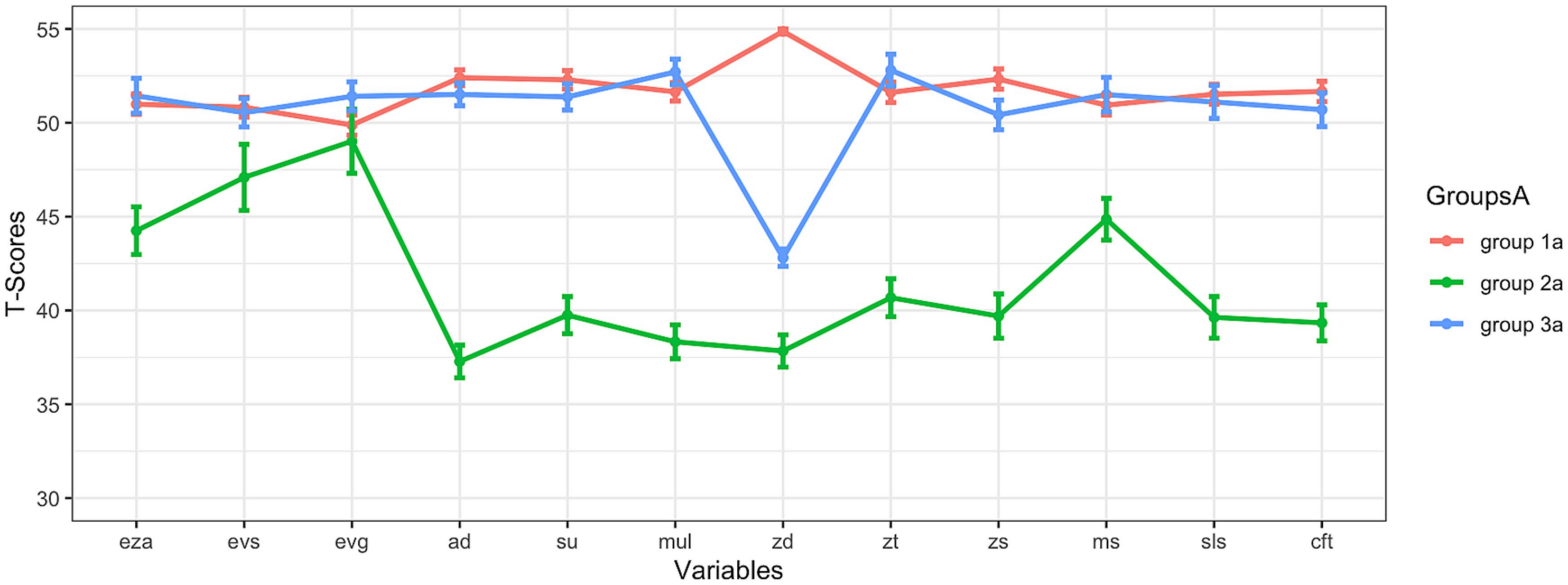
Results at subtest level (all variables were used for clustering). eza = dot enumeration; evs = symbolic magnitude comparison; evg = mixed magnitude comparison; ad = addition; su = subtraction; mul = multiplication; zd = transcoding; zt = number sets; zs = number line; ms = matrix span; sls = reading fluency; cft = intelligence; note: the means and standard errors are shown.

**Figure 2:**
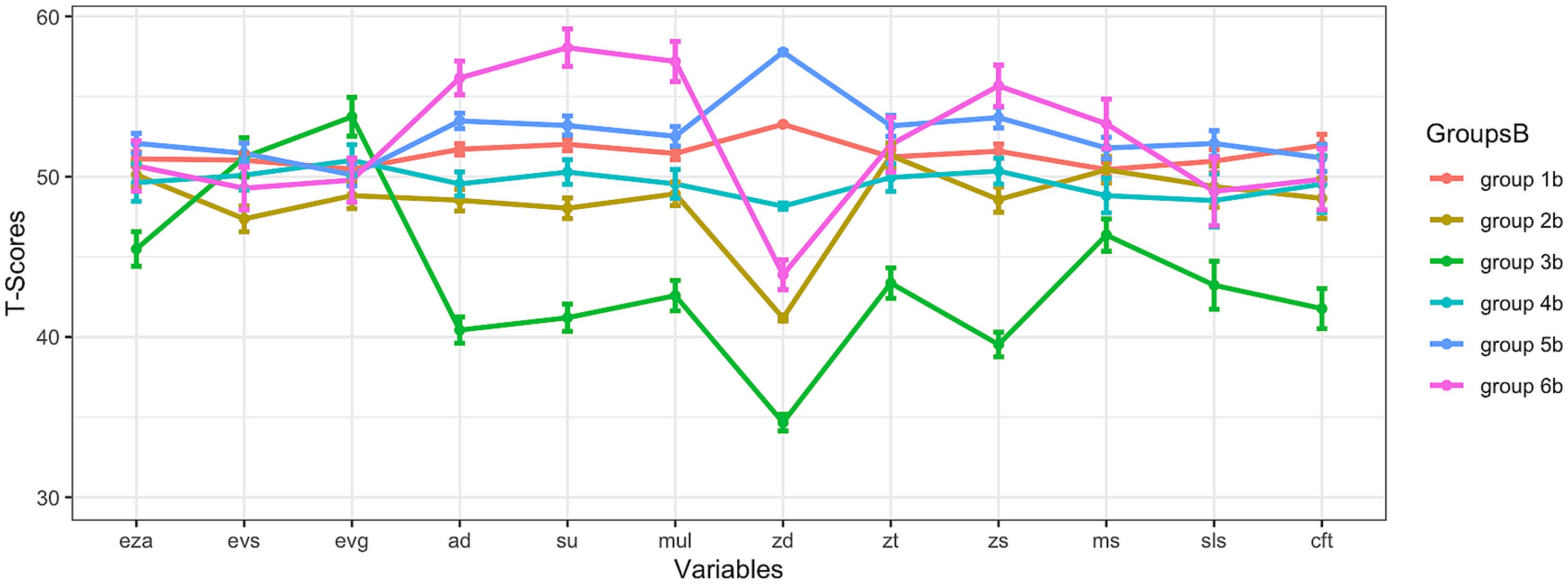
Results at subtest level (only mathematical variables were used for clustering). eza = dot enumeration; evs = symbolic magnitude comparison; evg = mixed magnitude comparison; ad = addition; su = subtraction; mul = multiplication; zd = transcoding; zt = number sets; zs = number line; ms = matrix span; sls = reading fluency; cft = intelligence; note: the means and standard errors are shown.

**Figure 3:**
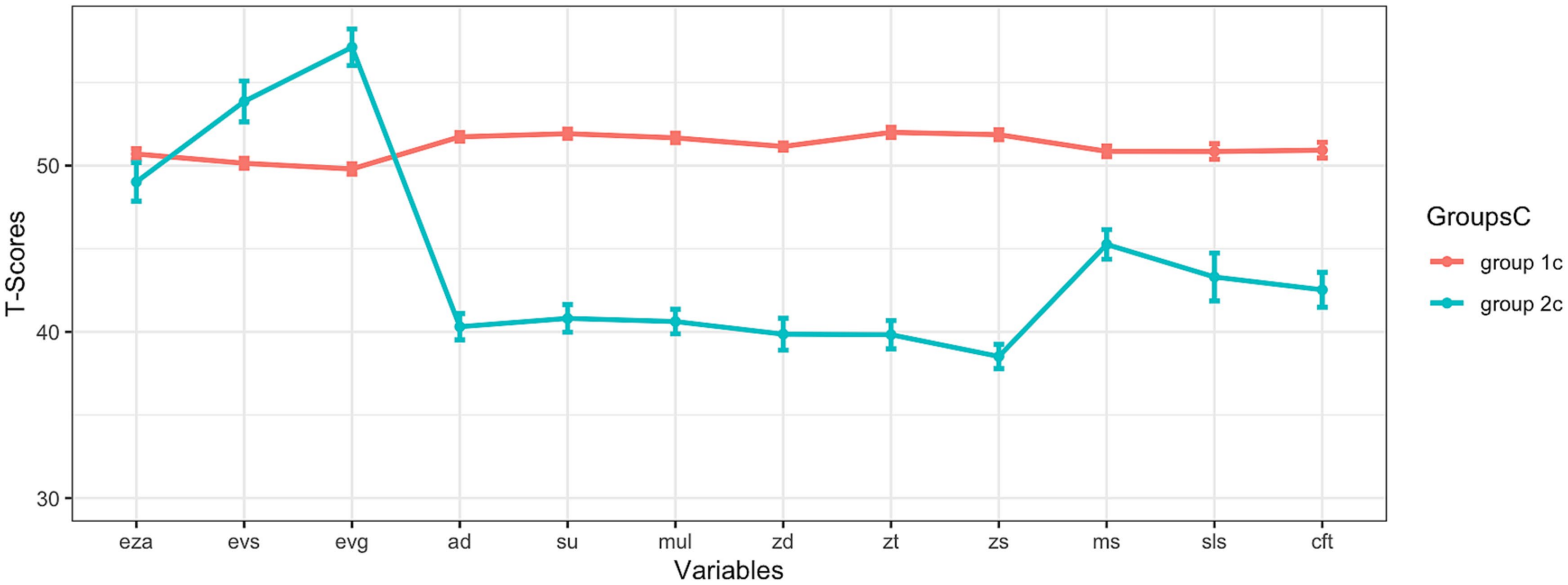
Results at construct level (only mathematical variables were used for clustering). eza = dot enumeration; evs = symbolic magnitude comparison; evg = mixed magnitude comparison; ad = addition; su = subtraction; mul = multiplication; zd = transcoding; zt = number sets; zs = number line; ms = matrix span; sls = reading fluency; cft = intelligence; note: the means and standard errors are shown.

Figures 1–3 present cognitive profiles. Each subsection of the results section discusses the figure that is relevant to the respective analysis. For example, Figure 1 is discussed in the first subsection of the results as it is about the results at subtest level, if all variables were used for clustering. These figures show *T*-scores, which have an overall mean of 50 and a standard deviation of 10. This means that scores below 50 are below average, and scores above 50 are above average. For each identified subgroup, the corresponding values are visualized to allow comparisons across subgroups. Exact values can be found in the tables.

The Supplementary materials include Supplementary Table A1 and Supplementary Figure A1, which present the descriptive statistics based on a division of the total sample into subgroups according to their results in the HRT 1–4 (PR > 16; PR < 16; PR < 10; PR < 5). This allows the comparison of the identified subtypes, as presented in the following subsections, with those groups of children that show a specific performance in the HRT 1–4.

### 3.1 Results at subtest level (all variables were used for clustering)

In this approach, all subtest-level results (except for the HRT 1–4) described in the methods section were used for subtyping, and incomplete data sets were excluded from the analysis ( $n_{2}\;$ = 478). The analyses revealed three subgroups: Their cognitive profiles are shown in Figure 1. This three-subgroup-solution with an absolute BIC of 41008.16 was the best solution to subdivide the children of the analyzed sample into subgroups based on data. The best fitting model (EEV) that was therefore used for clustering encompasses clusters with the following characteristics: ellipsoidal distributions with equal volume and equal shape (Scrucca et al., 2016; Fraley et al., 2024). This solution will be analyzed in more detail, now.

Figure 1 shows that the curves (cognitive profiles) of group 1a and group 3a were very similar and that the mean scores of both subgroups for the subtests often were close to the *T*-score of 50, i.e., to the expected overall mean value on population level for all children in general. Only regarding the variable transcoding, group 1a and group 3a seemed to differ strongly: Here, group 1a was clearly superior to group 3a. While group 1a showed an above-average mean for transcoding, the mean score of group 3a was even below the *T*-score of 45 for this variable. The mean scores of group 2a were below the *T*-score of 50 for each test. The curve that displays the cognitive profile of group 2a was always below the curves of the groups 1a and 3a. Descriptive results on the 3 subgroups (mean, standard deviation, and standard error) are shown in Table 1.

**Table 1:** Descriptive results (Clustering on subtest level, all variables were used for clustering).

Subtests	Group 1a			Group 2a			Group 3a		
	*M*	SD	SE	*M*	SD	SE	*M*	SD	SE
Dot enumeration	50.99	9.42	0.54	44.25	9.59	1.27	51.44	10.21	0.93
Symbolic magnitude comparison	50.83	9.23	0.53	47.09	13.30	1.76	50.54	8.28	0.76
Mixed magnitude comparison	49.88	9.41	0.54	49.02	12.90	1.71	51.41	8.44	0.77
Addition	52.40	7.25	0.42	37.28	6.60	0.87	51.51	6.61	0.60
Subtraction	52.29	8.53	0.49	39.75	7.51	0.99	51.38	7.67	0.70
Multiplication	51.65	8.45	0.49	38.33	6.76	0.90	52.71	7.40	0.68
Transcoding	54.87	2.13	0.12	37.84	6.47	0.86	42.81	4.99	0.46
Number sets	51.62	9.36	0.54	40.68	7.63	1.01	52.80	9.31	0.85
Number line	52.33	9.36	0.54	39.70	8.93	1.18	50.42	8.61	0.79
Matrix span	50.94	9.25	0.53	44.86	8.40	1.11	51.50	10.03	0.92
Reading fluency	51.52	9.26	0.53	39.63	8.38	1.11	51.11	9.64	0.88
Intelligence	51.67	9.39	0.54	39.34	7.21	0.96	50.70	9.84	0.90

Parallelism was tested for this three-subgroup-solution that is shown in Figure 1 in two ways. In a first step, all resulting subgroups were tested for parallelism in a joint analysis with *profileR* (Bulut and Desjardins, 2020; Bulut and Desjardins, 2022). As the result of this analysis became significant (*p* < 0.001), at least some cognitive profiles of the identified subgroups did not run parallel to each other. However, individual cognitive profiles of the subgroups could still have been run parallel to each other, while others did not. Therefore, in a second step, each subgroup was tested against every other subgroup individually to analyze whether there was evidence against parallelism. All of these analyses led to significant results (at least *p* < 0.001). Therefore, the data did not support parallelism for any profile comparison.

Group comparisons are shown in Table 2. As group 2a was significantly inferior to groups 1a and 3a in almost all areas, group 2a seemed to be considerably impaired. Only in some areas of basic numerical processing (*symbolic magnitude comparison* and *mixed magnitude comparison*) the mentioned differences between group 1a and group 2a, respectively, group 2a and group 3a were not always significant, whereas Bayesian analyses actually suggested equality only between group 1a and group 2a regarding the variable *mixed magnitude comparison* (BF = 0.17). Regarding the non-significant differences between group 2a and group 3a, the Bayesian analyses did not clearly confirm that there was equality because the BF was above 0.33. Significant difference between groups 1a and 3a was found in transcoding, only. In fact, if comparing group 1a and 3a there were non-significant differences in most subtests and Bayesian analyses even showed that equality has to be assumed in many cases because the BF was below 0.33, often. The different transcoding abilities seemed to be decisive for the differentiation between group 1a and group 3a.

**Table 2:** Group comparison for the resulting subgroups (clustered on subtest level, all variables were used for clustering).

Subtests	Group 1a vs. 2a			Group 1a vs. 3a			Group 2a vs. 3a		
	Tukey	BF	*d*	Tukey	BF	*d*	Tukey	BF	*d*
Dot enumeration	6.75***	1.17*10^4^	0.71	−0.45	0.13	0.05	−7.20***	1.22*10^3^	0.72
Symbolic magnitude comparison	3.75*	1.08	0.38	0.29	0.12	0.03	−3.45	0.77	0.34
Mixed magnitude comparison	0.86	0.17	0.09	−1.53	0.38	0.17	−2.39	0.37	0.34
Addition	15.12***	5.13*10^34^	2.11	0.89	0.23	0.13	−14.23***	1.62*10^25^	2.15
Subtraction	12.53***	1.50*10^22^	1.50	0.91	0.20	0.11	−11.63***	2.45*10^14^	1.53
Multiplication	13.31***	4.51*10^28^	1.62	−1.06	0.26	0.13	−14.38***	2.69*10^22^	2.00
Transcoding	17.03***	9.32*10^54^	5.28	12.06***	4.60*10^83^	3.76	−4.97***	1.74*10^4^	0.90
Number sets	10.94***	1.55*10^16^	1.20	−1.18	0.23	0.13	−12.12***	3.48*10^13^	1.38
Number line	12.62***	5.73*10^15^	1.36	1.91	0.71	0.21	−10.72***	4.70*10^9^	1.23
Matrix span	6.08***	2.83*10^3^	0.67	−0.56	0.14	0.06	−6.64***	2.12*10^3^	0.70
Reading fluency	11.89***	3.40*10^14^	1.30	0.42	0.13	0.04	−11.48***	6.84*10^9^	1.24
Intelligence	12.33***	9.04*10^21^	1.36	0.97	0.18	0.10	−11.36***	6.34*10^10^	1.25

*p < 0.05; **p < 0.01; ***p < 0.001.

There was a disproportionate accumulation of children with dyscalculia or arithmetic difficulties in subgroup 2a (58.93% had a PR below 16, 55.36% had a PR below 10, and 26.79% had even a PR below 5): more than half of the individuals (if cut-off scores of PR < 16 or PR < 10 were applied) in this subgroup were dyscalculic. No group consisted solely of either dyscalculic or non-dyscalculic children. However, the proportion of dyscalculic children in group 2a was very high. The *χ*^2^-Tests confirmed that the proportion of dyscalculic children was not equally distributed among the subgroups (if PR < 16: *χ*^2^ =87.30, *p* < 0.001, Cramér’s *V* = 0.40; if PR < 10: *χ*^2^ = 103.73, *p* < 0.001, Cramér’s *V* = 0.47; if PR < 5: *χ*^2^ = 54.02, *p* < 0.001, Cramér’s *V* = 0.34). The results for Fisher’s exact test to check the results of the *χ*^2^-tests for robustness were almost identical and therefore robust. If the exact distributions of dyscalculic versus non-dyscalculic children across the groups (based on the PR) are of interest, these can be found in Supplementary Table A2.

### 3.2 Results at subtest level (only mathematical variables were used for clustering)

In this approach, only the results from the mathematical subtests of the CODY-M 2–4 (Kuhn et al., 2017) were used for subtyping ( $n_{1}$ = 1,015). The analysis revealed six subgroups, which are shown in Figure 2. This six-subgroup-solution with an absolute BIC of 63700.88 was the best solution to subdivide children of the analyzed sample into subgroups based on data: The best fitting model (EVE) that was therefore used for clustering encompasses clusters with the following characteristics: ellipsoidal distributions with equal volume and equal orientation (Scrucca et al., 2016; Fraley et al., 2024). This solution will be analyzed in more detail, now.

If looking at the different curves which reflect cognitive profiles, it was striking that especially group 3b laid below the curves of all the other groups (except for two variables, which belong to the construct *basic numerical processing* (BNP): *symbolic magnitude comparison* and *mixed magnitude comparison*). The graph of group 4b stood out because the mean values for the individual variables fluctuated only weakly around the T-scores of 50. Children in this subgroup thus seemed to have predominantly average scores and were neither high- nor low-performers. Even though the graph of group 2b was similar to the graph of group 4b, the mean values of group 2b were usually somewhat lower. The graph of group 1b was almost constantly slightly above the graph of group 4b and the T-scores were slightly above 50. The graph of group 5b was similar to the graph of group 1b, but the children of group 5b tended to perform slightly better on average than the children of group 1b. In transcoding, group 5b showed the highest scores of all groups. Group 6b showed a very heterogeneous competence profile: in BNP, group 6b’s scores were in the average range; the calculations skills of group 6b seemed to be very high because the children of group 6b reached the highest scores of all groups in this area. But transcoding skills of group 6b were lower than the transcoding skills of the (other) groups that tended to perform above average in other subtests: group 6b’s mean score in transcoding was below the *T*-score of 45. In intelligence and reading ability, group 6b also appeared to be in the average performance range. Descriptive results on the 6 subgroups (mean, standard deviation, and standard error) are shown in Table 3.

**Table 3:** Descriptive results (Clustering on subtest level; mathematical variables were used for clustering).

Subtests	Group 1b			Group 2b			Group 3b			Group 4b			Group 5b			Group 6b		
	***M***	**SD**	**SE**	***M***	**SD**	**SE**	***M***	**SD**	**SE**	***M***	**SD**	**SE**	***M***	**SD**	**SE**	***M***	**SD**	**SE**
Dot enumeration	51.11	9.72	0.45	50.12	9.25	0.79	45.49	10.04	1.08	49.63	10.58	1.17	52.08	8.79	0.62	50.67	10.26	1.57
Symbolic magnitude comparison	51.03	9.58	0.44	47.37	9.49	0.81	51.21	11.31	1.22	50.09	8.47	0.94	51.46	9.00	0.64	49.28	8.77	1.34
Mixed magnitude comparison	50.47	9.48	0.44	48.82	9.49	0.81	53.74	11.19	1.21	51.02	8.87	0.98	50.11	9.66	0.69	49.79	8.95	1.37
Addition	51.71	7.61	0.35	48.53	7.83	0.67	40.43	7.59	0.82	49.55	6.83	0.75	53.48	6.81	0.48	56.16	6.87	1.05
Subtraction	52.02	8.48	0.39	48.03	7.57	0.64	41.20	7.90	0.85	50.29	7.00	0.77	53.19	8.40	0.60	58.05	7.64	1.17
Multiplication	51.45	8.24	0.38	48.93	8.52	0.73	42.58	8.82	0.95	49.55	8.18	0.90	52.52	8.58	0.61	57.19	8.22	1.25
Transcoding	53.27	0.44	0.02	41.18	2.09	0.18	34.66	4.84	0.52	48.16	1.78	0.20	57.78	0.89	0.06	43.88	6.09	0.93
Number sets	51.23	9.67	0.45	51.32	9.25	0.79	43.36	8.83	0.95	49.95	7.85	0.87	53.17	9.18	0.65	52.00	11.22	1.71
Number line	51.59	9.63	0.44	48.57	9.32	0.79	39.53	7.12	0.77	50.35	7.32	0.81	53.69	9.20	0.65	55.67	8.44	1.29
Matrix span	50.44	9.54	0.44	50.43	9.64	0.82	46.36	9.33	1.01	48.82	9.81	1.08	51.79	9.41	0.67	53.30	10.07	1.54
Reading fluency	50.97	9.39	0.72	49.38	10.69	1.29	43.23	10.31	1.50	48.51	10.28	1.65	52.07	9.24	0.80	49.08	9.78	2.13
Intelligence	51.96	9.06	0.68	48.65	10.57	1.26	41.77	8.57	1.25	49.54	11.14	1.78	51.17	9.76	0.84	49.84	8.95	1.91

Parallelism was tested for this six-subgroup-solution that is shown in Figure 2 in two ways. In a first step, all resulting subgroups were tested for parallelism in a joint analysis with *profileR* (Bulut and Desjardins, 2020; Bulut and Desjardins, 2022). As the result of this analysis became significant (*p* < 0.001), at least some cognitive profiles of the identified subgroups did not run parallel to each other. However, individual cognitive profiles of the subgroups could still have been run parallel to each other, while others did not. Therefore, in a second step, each subgroup was tested against every other subgroup individually to analyze whether there was evidence for parallelism. All of these analyses led to significant results (at least *p* < 0.01). Therefore, the data did not support parallelism for any direct profile comparison.

Group comparisons are shown in Tables 4–9. In Table 6 is shown that group 3b was consistently (and often significantly) inferior to all other groups in all mathematical tests (except for the subtests that can be assigned to the BNP: *dot enumeration*, *symbolic magnitude comparison*, *mixed magnitude comparison*). Earlier, it was described that the graph of group 2b laid slightly, but noticeable below the graphs of the other groups in most areas (except of group 3b). In Table 5, it can be seen that many of these differences became significant, and also with Bayesian analyses, evidence emerged that group 2b showed reduced performance in many subtests compared to the groups 1b, 5b, and 6b (especially in mathematical subdomains). Although group 4b tended to show higher performance descriptively compared to group 2b (Table 5), most of these differences did not become significant, and in some subtests, due to the fact that BFs were below 0.33, their performance seemed equal. Even though group 2b and group 4b were hardly distinguishable in many subtests because the differences in these subtests were not significant, they still seemed to be separable groups due to the visually different curves in the coordinate system and the significant difference in transcoding (*p* < 0.001; BF = 5.87*10^65). The performance of group 4b seemed to be slightly better than the performance of group 2b in 8 of 12 subtests (the exceptions are *dot enumeration*, *number sets, matrix span* and *reading fluency*, although these differences did not become significant): Therefore, group 4b seemed less impaired if these two groups were compared with each other.

**Table 4:** Comparison between subgroup 1b and the other subgroups (clustered on subtest level; mathematical variables were used for clustering).

Subtests	**Group 1b**														
	**Vs. Group 2b**			**Vs. Group 3b**			**Vs. Group 4b**			**Vs. Group 5b**			**Vs. Group 6b**		
	**Tukey**	**BF**	***d***	**Tukey**	**BF**	***d***	**Tukey**	**BF**	***d***	**Tukey**	**BF**	***d***	**Tukey**	**BF**	***d***
Dot enumeration	1.00	0.19	0.10	5.62***	1.06*10^4^	0.58	1.48	0.28	0.15	−0.97	0.19	0.10	0.44	0.18	0.04
Symbolic magnitude comparison	3.66**	1.91*10^2^	0.38	−0.18	0.13	0.02	0.94	0.18	0.10	−0.43	0.11	0.05	1.75	0.32	0.18
Mixed magnitude comparison	1.65	0.51	0.17	−3.27*	2.83	0.34	−0.55	0.15	0.06	0.36	0.10	0.04	0.68	0.19	0.07
Addition	3.18***	6.95*10^2^	0.41	11.28***	8.09*10^28^	1.48	2.16	2.04	0.29	−1.78	6.79	0.24	−4.46**	94.70	0.59
Subtraction	3.99***	6.38*10^4^	0.48	10.82***	1.74*10^22^	1.29	1.72	0.86	0.21	−1.17	0.34	0.14	−6.03***	1.87*10^3^	0.72
Multiplication	2.52*	11.83	0.30	8.87***	2.01*10^15^	1.06	1.90	0.77	0.23	−1.07	0.29	0.13	−5.74***	1.11*10^3^	0.70
Transcoding	12.09***	1.78*10^279^	11.32	18.60***	9.18*10^140^	9.58	5.11***	6.15*10^92^	6.41	−4.51***	1.22*10^296^	7.38	9.38***	5.25*10^18^	5.21
Number sets	−0.09	0.11	0.01	7.87***	1.12*10^9^	0.82	1.28	0.30	0.14	−1.93	1.53	0.20	−0.77	0.19	0.08
Number line	3.02**	17.97	0.32	12.05***	8.96*10^32^	1.30	1.23	0.31	0.13	−2.10	2.55	0.22	−4.09	4.81	0.43
Matrix span	0.01	0.11	0.00	4.08**	72.09	0.43	1.62	0.34	0.17	−1.35	0.37	0.14	−2.86	0.87	0.30
Reading fluency	1.59	0.28	0.16	7.74***	7.27*10^3^	0.81	2.46	0.49	0.26	−1.10	0.21	0.81	1.88	0.33	0.20
Intelligence	3.31	2.59	0.35	10.19***	1.59*10^8^	1.14	2.42	0.49	0.26	0.79	0.16	0.08	2.12	0.37	0.23

*p < 0.05; **p < 0.01; ***p < 0.001.

**Table 5:** Comparison between subgroup 2b and the other subgroups (clustered on subtest level; mathematical variables were used for clustering).

Subtests	**Group 2b**														
	**Vs. Group 1b**			**Vs. Group 3b**			**Vs. Group 4b**			**Vs. Group 5b**			**Vs. Group 6b**		
	**Tukey**	**BF**	***d***	**Tukey**	**BF**	***d***	**Tukey**	**BF**	***d***	**Tukey**	**BF**	***d***	**Tukey**	**BF**	***d***
Dot enumeration	−1.00	0.19	0.10	4.63**	45.78	0.48	0.48	0.16	0.05	−1.96	0.78	0.22	−0.56	0.20	0.06
Symbolic magnitude comparison	−3.66**	1.91*10^2^	0.38	−3.84*	4.82	0.38	−2.72	1.27	0.30	−4.10**	2.38*10^2^	0.44	−1.91	0.35	0.20
Mixed magnitude comparison	−1.65	0.51	0.17	−4.93**	45.62	0.48	−2.21	0.59	0.24	−1.29	0.25	0.13	−0.97	0.22	0.10
Addition	−3.18***	6.95*10^2^	0.41	8.10***	8.59*10^9^	1.05	−1.02	0.24	0.14	−4.96***	2.03*10^6^	0.68	−7.63***	2.71*10^5^	1.00
Subtraction	−3.99***	6.38*10^4^	0.48	6.83***	1.36*10^7^	0.89	−2.26	1.46	0.31	−5.16***	9.59*10^5^	0.64	−10.02***	3.11*10^9^	1.32
Multiplication	−2.52*	11.83	0.30	6.35***	5.73*10^4^	0.74	−0.61	0.17	0.07	−3.59**	1.03*10^2^	0.42	−8.25***	1.36*10^5^	0.98
Transcoding	−12.09***	1.78*10^279^	11.32	6.52***	1.17*10^22^	1.91	−6.98***	5.87*10^65^	3.53	−16.60***	3.90*10^228^	11.06	−2.70***	7.37	0.78
Number sets	0.09	0.11	0.01	7.96***	8.41*10^6^	0.88	1.37	0.29	0.16	−1.85	0.58	0.20	−0.68	0.20	0.07
Number line	−3.02**	17.97	0.32	9.03***	2.52*10^11^	1.06	−1.79	0.49	0.21	−5.12***	1.35*10^4^	0.55	−7.11***	1.22*10^3^	0.78
Matrix span	−0.01	0.11	0.00	4.07*	13.14	0.43	1.61	0.29	0.17	−1.36	0.27	0.14	−2.87	0.68	0.30
Reading fluency	−1.59	0.28	0.16	6.15*	13.00	0.58	0.87	0.23	0.08	−2.69	0.80	0.28	0.29	0.26	0.03
Intelligence	−3.31	2.59	0.35	6.88**	79.01	0.70	−0.89	0.23	0.08	−2.52	0.62	0.25	−1.19	0.28	0.12

*p < 0.05; **p < 0.01; ***p < 0.001.

**Table 6:** Comparison between subgroup 3b and the other subgroups (clustered on subtest level; mathematical variables were used for clustering).

Subtests	**Group 3b**														
	**Vs. Group 1b**			**Vs. Group 2b**			**Vs. Group 4b**			**Vs. Group 5b**			**Vs. Group 6b**		
	**Tukey**	**BF**	***d***	**Tukey**	**BF**	***d***	**Tukey**	**BF**	***d***	**Tukey**	**BF**	***d***	**Tukey**	**BF**	***d***
Dot enumeration	−5.62***	1.06*10^4^	0.58	−4.63**	45.78	0.48	−4.15	3.73	0.40	−6.59***	4.63*10^4^	0.72	−5.19*	5.58	0.51
Symbolic magnitude comparison	0.18	0.13	0.02	3.84*	4.82	0.38	1.12	0.21	0.11	−0.26	0.14	0.03	1.93	0.33	0.18
Mixed magnitude comparison	3.27*	2.83	0.34	4.93**	45.62	0.48	2.72	0.68	0.27	3.63*	3.50	0.36	3.95	1.62	0.38
Addition	−11.28***	8.09*10^28^	1.48	−8.10***	8.59*10^9^	1.05	−9.12***	7.60*10^10^	1.26	−13.05***	7.31*10^31^	1.85	−15.73***	1.03*10^18^	2.14
Subtraction	−10.82***	1.74*10^22^	1.29	−6.83***	1.36*10^7^	0.89	−9.10***	1.54*10^10^	1.22	−11.99***	1.67*10^21^	1.45	−16.85***	1.82*10^18^	2.16
Multiplication	−8.87***	2.01*10^15^	1.06	−6.35***	5.73*10^4^	0.74	−6.97***	3.57*10^4^	0.82	−9.94***	6.36*10^13^	1.15	−14.60***	2.38*10^12^	1.69
Transcoding	−18.60***	9.18*10^140^	9.58	−6.52***	1.17*10^22^	1.91	−13.50***	3.72*10^52^	3.67	−23.11***	1.16*10^124^	8.38	−9.22***	2.75*10^11^	1.75
Number sets	−7.87***	1.12*10^9^	0.82	−7.96***	8.41*10^6^	0.88	−6.59***	1.54*10^4^	0.79	−9.81***	1.90*10^12^	1.08	−8.64***	8.49*10^2^	0.89
Number line	−12.05***	8.96*10^32^	1.30	−9.03***	2.52*10^11^	1.06	−10.82***	6.55*10^14^	1.50	−14.15***	6.88*10^30^	1.64	−16.14***	8.17*10^17^	2.13
Matrix span	−4.08**	72.09	0.43	−4.07*	13.14	0.43	−2.46	0.60	0.26	−5.43***	1.46*10^3^	0.58	−6.94**	1.37*10^2^	0.72
Reading fluency	−7.74***	7.27*10^3^	0.81	−6.15*	13.00	0.58	−5.28	2.51	0.51	−8.84***	7.72*10^4^	0.93	−5.85	1.93	0.58
Intelligence	−10.19***	1.59*10^8^	1.14	−6.88**	79.01	0.70	−7.77**	46.73	0.79	−9.40***	4.69*10^5^	0.99	−8.07*	45.89	0.93

*p < 0.05; **p < 0.01; ***p < 0.001.

**Table 7:** Comparison between subgroup 4b and the other subgroups (clustered on subtest level; mathematical variables were used for clustering).

Subtests	**Group 4b**														
	**Vs. Group 1b**			**Vs. Group 2b**			**Vs. Group 3b**			**Vs. Group 5b**			**Vs. Group 6b**		
	**Tukey**	**BF**	***d***	**Tukey**	**BF**	***d***	**Tukey**	**BF**	***d***	**Tukey**	**BF**	***d***	**Tukey**	**BF**	***d***
Dot enumeration	−1.48	0.28	0.15	−0.48	0.16	0.05	4.15	3.73	0.40	−2.45	0.71	0.26	−1.04	0.23	0.10
Symbolic magnitude comparison	−0.94	0.18	0.10	2.72	1.27	0.30	−1.12	0.21	0.11	−1.38	0.28	0.16	0.81	0.22	0.09
Mixed magnitude comparison	0.55	0.15	0.06	2.21	0.59	0.24	−2.72	0.68	0.27	0.91	0.19	0.10	1.23	0.25	0.14
Addition	−2.16	2.04	0.29	1.02	0.24	0.14	9.12***	7.60*10^10^	1.26	−3.94***	1.06*10^3^	0.58	−6.61***	1.28*10^4^	0.97
Subtraction	−1.72	0.86	0.21	2.26	1.46	0.31	9.10***	1.54*10^10^	1.22	−2.89	8.59	0.36	−7.75***	1.36*10^5^	1.07
Multiplication	−1.90	0.77	0.23	0.61	0.17	0.07	6.97***	3.57*10^4^	0.82	−2.97	4.06	0.35	−7.64***	6.29*10^3^	0.93
Transcoding	−5.11***	6.15*10^92^	6.41	6.98***	5.87*10^65^	3.53	13.50***	3.72*10^52^	3.67	−9.62***	1.07*10^129^	7.89	4.27***	1.17*10^3^	1.11
Number sets	−1.28	0.30	0.14	−1.37	0.29	0.16	6.59***	1.54*10^4^	0.79	−3.22	5.32	0.37	−2.05	0.33	0.22
Number line	−1.23	0.31	0.13	1.79	0.49	0.21	10.82***	6.55*10^14^	1.50	−3.33	17.29	0.38	−5.32*	68.16	0.69
Matrix span	−1.62	0.34	0.17	−1.61	0.29	0.17	2.46	0.60	0.26	−2.97	2.02	0.31	−4.49	2.61	0.45
Reading fluency	−2.46	0.49	0.26	−0.87	0.23	0.08	5.28	2.51	0.51	−3.56	1.31	0.38	−0.57	0.28	0.06
Intelligence	−2.42	0.49	0.26	0.89	0.23	0.08	7.77**	46.73	0.79	−1.63	0.28	0.16	−0.30	0.27	0.03

*p < 0.05; **p < 0.01; ***p < 0.001.

**Table 8:** Comparison between subgroup 5b and the other subgroups (clustered on subtest level; mathematical variables were used for clustering).

Subtests	**Group 5b**														
	**Vs. Group 1b**			**Vs. Group 2b**			**Vs. Group 3b**			**Vs. Group 4b**			**Vs. Group 6b**		
	**Tukey**	**BF**	***d***	**Tukey**	**BF**	***d***	**Tukey**	**BF**	***d***	**Tukey**	**BF**	***d***	**Tukey**	**BF**	***d***
Dot enumeration	0.97	0.19	0.10	1.96	0.78	0.22	6.59***	4.63*10^4^	0.72	2.45	0.71	0.26	1.41	0.25	0.16
Symbolic magnitude comparison	0.43	0.11	0.05	4.10**	2.38*10^2^	0.44	0.26	0.14	0.03	1.38	0.28	0.16	2.19	0.47	0.24
Mixed magnitude comparison	−0.36	0.10	0.04	1.29	0.25	0.13	−3.63*	3.50	0.36	−0.91	0.19	0.10	0.32	0.18	0.03
Addition	1.78	6.79	0.24	4.96***	2.03*10^6^	0.68	13.05***	7.31*10^31^	1.85	3.94***	1.06*10^3^	0.58	−2.68	2.15	0.39
Subtraction	1.17	0.34	0.14	5.16***	9.59*10^5^	0.64	11.99***	1.67*10^21^	1.45	2.89	8.59	0.36	−4.86**	44.56	0.59
Multiplication	1.07	0.29	0.13	3.59**	1.03*10^2^	0.42	9.94***	6.36*10^13^	1.15	2.97	4.06	0.35	−4.67*	21.99	0.55
Transcoding	4.51***	1.22*10^296^	7.38	16.60***	3.90*10^228^	11.06	23.11***	1.16*10^124^	8.38	9.62***	1.07*10^129^	7.89	13.89***	3.13*10^32^	5.19
Number sets	1.93	1.53	0.20	1.85	0.58	0.20	9.81***	1.90*10^12^	1.08	3.22	5.32	0.37	1.17	0.22	0.12
Number line	2.10	2.55	0.22	5.12***	1.35*10^4^	0.55	14.15***	6.88*10^30^	1.64	3.33	17.29	0.38	−1.99	0.39	0.22
Matrix span	1.35	0.37	0.14	1.36	0.27	0.14	5.43***	1.46*10^3^	0.58	2.97	2.02	0.31	−1.51	0.27	0.16
Reading fluency	1.10	0.21	0.81	2.69	0.80	0.28	8.84***	7.72*10^4^	0.93	3.56	1.31	0.38	2.98	0.54	0.32
Intelligence	−0.79	0.16	0.08	2.52	0.62	0.25	9.40***	4.69*10^5^	0.99	1.63	0.28	0.16	1.33	0.28	0.14

*p < 0.05; **p < 0.01; ***p < 0.001.

**Table 9:** Comparison between subgroup 6b and the other subgroups (clustered on subtest level; mathematical variables were used for clustering).

Subtests	**Group 6b**														
	**Vs. Group 1b**			**Vs. Group 2b**			**Vs. Group 3b**			**Vs. Group 4b**			**Vs. Group 5b**		
	**Tukey**	**BF**	***d***	**Tukey**	**BF**	***d***	**Tukey**	**BF**	***d***	**Tukey**	**BF**	***d***	**Tukey**	**BF**	***d***
Dot enumeration	−0.44	0.18	0.04	0.56	0.20	0.06	5.19 *	5.58	0.51	1.04	0.23	0.10	−1.41	0.25	0.16
Symbolic magnitude comparison	−1.75	0.32	0.18	1.91	0.35	0.20	−1.93	0.33	0.18	−0.81	0.22	0.09	−2.19	0.47	0.24
Mixed magnitude comparison	−0.68	0.19	0.07	0.97	0.22	0.10	−3.95	1.62	0.38	−1.23	0.25	0.14	−0.32	0.18	0.03
Addition	4.46**	94.70	0.59	7.63***	2.71*10^5^	1.00	15.73***	1.03*10^18^	2.14	6.61***	1.28*10^4^	0.97	2.68	2.15	0.39
Subtraction	6.03***	1.87*10^3^	0.72	10.02***	3.11*10^9^	1.32	16.85***	1.82*10^18^	2.16	7.75***	1.36*10^5^	1.07	4.86**	44.56	0.59
Multiplication	5.74***	1.11*10^3^	0.70	8.25***	1.36*10^5^	0.98	14.60***	2.38*10^12^	1.69	7.64***	6.29*10^3^	0.93	4.67*	21.99	0.55
Transcoding	−9.38***	5.25*10^18^	5.21	2.70***	7.37	0.78	9.22***	2.75*10^11^	1.75	−4.27***	1.17*10^3^	1.11	−13.89***	3.13*10^32^	5.19
Number sets	0.77	0.19	0.08	0.68	0.20	0.07	8.64***	8.49*10^2^	0.89	2.05	0.33	0.22	−1.17	0.22	0.12
Number line	4.09	4.81	0.43	7.11***	1.22*10^3^	0.78	16.14***	8.17*10^17^	2.13	5.32*	68.16	0.69	1.99	0.39	0.22
Matrix span	2.86	0.87	0.30	2.87	0.68	0.30	6.94**	1.37*10^2^	0.72	4.49	2.61	0.45	1.51	0.27	0.16
Reading fluency	−1.88	0.33	0.20	−0.29	0.26	0.03	5.85	1.93	0.58	0.57	0.28	0.06	−2.98	0.54	0.32
Intelligence	−2.12	0.37	0.23	1.19	0.28	0.12	8.07*	45.89	0.93	0.30	0.27	0.03	−1.33	0.28	0.14

*p < 0.05; **p < 0.01; ***p < 0.001.

There was a disproportionately large number of children with dyscalculia in group 3b (39.76% had a PR below 16, 36.14% had a PR below 10 and 21.69% had even a PR below 5). However, there was no group in which there was no child with dyscalculia, if cut-off scores of PR < 16 or PR < 10 were applied – but if a cut-off score of PR < 5 was applied, there was no dyscalculic child in group 4b or 6b. Besides group 3b, larger accumulations of children with dyscalculia were also found in group 2b (18.25% had a PR below 16, 14.60% had a PR below 10 and 5.84% had even a PR below 5) and group 4b (16.05% had a PR below 16, 12.35% had a PR below 10, but no child of this subgroup had a PR below 5). This comparison of group 2b and 4b supports the previously stated assumption that group 4b seemed to encompass less impaired children than group 2b did. The *χ*^2^-tests showed that the proportion of dyscalculic children was not equally distributed among the subgroups (if PR < 16: *χ*^2^ = 79.78, *p* < 0.001, Cramér’s *V* = 0.28; if PR < 10: *χ*^2^ = 95.06, *p* < 0.001, Cramér’s *V* = 0.31; if PR < 5: *χ*^2^ = 80.09, *p* < 0.001, Cramér’s *V* = 0.28). The results for Fisher’s exact test to check the results of *χ*^2^-tests for robustness were almost identical and therefore robust. If the exact distributions of dyscalculic versus non-dyscalculic children across the groups (based on the PR) are of interest, these can be found in the electronic supplements (Supplementary Table A3).

### 3.3 Results at construct level (both approaches)

When the analyses were conducted at the construct level and all variables were considered, no meaningful subgroups were detected. Thus, it was not possible to divide the children/ their cognitive profiles into subgroups in a meaningful way using the selected analysis approach. This means that children with and without dyscalculia were not distinguishable in this approach.

But when only mathematical variables were considered to subtype at the construct level, two subgroups were identified. This two-subgroup-solution with an absolute BIC of 19858.66 was the best solution to subdivide children of the analyzed sample into subgroups based on data: The best fitting model (EEE) that was therefore used for clustering encompasses clusters with the following characteristics: ellipsoidal distributions with equal volume, shape, and orientation (Scrucca et al., 2016; Fraley et al., 2024). This solution will be analyzed in more detail, now.

If looking at the two resulting curves which reflect cognitive profiles, it was striking that the means of group 2c laid below the means of group 1c (except for two variables, which belong to the construct *BNP*: *symbolic magnitude comparison* and *mixed magnitude comparison*). In *symbolic magnitude comparison* and *mixed magnitude comparison*, group 2c showed higher scores than group 1c, even though group 2c otherwise appeared inferior to group 1c in the mathematical domain. In dot enumeration, the two groups were very close to each other. It is noticeable that group 1c had mean scores that fluctuated around the T-score of 50 or were often slightly above 50. Although the mean scores of group 2c in matrix span, reading fluency, and intelligence were higher than in the tasks reflecting calculation or CNP, the mean scores of group 2c in these three subareas (matrix span, reading fluency, intelligence) were still lower than those of group 1c. Descriptive results on the 2 subgroups (mean, standard deviation, and standard error) are shown in Table 10.

**Table 10:** Descriptive results and group comparisons (Clustering on construct level, mathematical variables were used for clustering).

Subtests	**Group 1c**			**Group 2c**			**Group 1c vs. Group 2c**		
	***M***	**SD**	**SE**	***M***	**SD**	**SE**	***t*-test**	**BF**	**d**
Dot enumeration	50.70	9.57	0.32	49.02	11.15	1.16	1.41	0.31	0.31
Symbolic magnitude comparison	50.14	9.24	0.30	53.86	11.91	1.23	−2.92**	7.20	0.39
Mixed magnitude comparison	49.80	9.30	0.31	57.12	10.61	1.10	−6.41***	3.62*10^7^	0.78
Addition	51.73	7.51	0.25	40.31	7.64	0.79	13.94***	9.94*10^36^	1.52
Subtraction	51.92	8.32	0.27	40.81	8.02	0.83	12.30***	1.20*10^29^	1.34
Multiplication	51.67	8.40	0.28	40.62	7.15	0.74	13.95***	1.05*10^37^	1.33
Transcoding	51.15	6.23	0.21	39.86	9.28	0.96	11.48***	2.49*10^25^	1.72
Number sets	52.00	9.10	0.30	39.83	8.23	0.85	13.46***	3.80*10^34^	1.35
Number line	51.86	9.23	0.30	38.52	7.09	0.73	16.78***	1.65*10^52^	1.47
Matrix span	50.86	9.61	0.32	45.26	8.57	0.89	5.41***	1.34*10^5^	0.59
Reading fluency	50.85	9.61	0.47	43.30	10.56	1.44	5.38***	9.68*10^4^	0.78
Intelligence	50.93	9.87	0.47	42.53	7.74	1.05	7.27***	4.20*10^9^	0.87

*p < 0.05; **p < 0.01; ***p < 0.001.

Parallelism was tested for this two-subgroup-solution that is shown in Figure 3. As the result of this analysis with *profileR* (Bulut and Desjardins, 2020; Bulut and Desjardins, 2022) became significant (*p* < 0.001), the cognitive profiles of the identified subgroups did not run parallel to each other.

Group comparisons are shown in Table 10. Group 1c and group 2c differed significantly from each other in all areas except for dot enumeration. In all subtests that belong to the constructs *calculation* and *CNP* – as well as in the domain-general subareas as matrix span, reading fluency, and intelligence – group 1c outperformed group 2c. However, in *symbolic magnitude comparison* and *mixed magnitude comparison*, group 2c achieved significantly higher results. The significant frequentist t-test results are supported by the Bayesian analyses, indicating the robustness of these results. Only for dot enumeration there was no significant difference apparent – the Bayesian analyses suggested equality, as the BF lays below 0.33.

There were a disproportionately large number of children with dyscalculia in group 2c (42.22% had a PR below 16, 37.78% had a PR below 10 and 21.11% had even a PR below 5). In comparison, children of group 1c were less likely to be dyscalculic (9.41% had a PR below 16, 6.53% had a PR below 10 and only 2.33% had a PR below 5). The *χ*^2^-Tests showed that the proportion of dyscalculic children was not equally distributed among the subgroups (if PR < 16: *χ*^2^ = 78.18, *p* < 0.001, Cramér’s *V* = 0.28; if PR < 10: *χ*^2^ = 90.48, *p* < 0.001, Cramér’s *V* = 0.30; if PR < 5: *χ*^2^ = 69.93, *p* < 0.001, Cramér’s *V* = 0.27). The results for Fisher’s exact test to check the results of the *χ*^2^-tests for robustness were almost identical and therefore robust. If the exact distributions of dyscalculic versus non-dyscalculic children across the groups (based on the PR) are of interest, these can be found in Supplementary Table A4.

## 4 Discussion

The purpose of this study was to answer the following question: To what extent do children with dyscalculia form a homogeneous group that can be distinguished from children without dyscalculia? To answer this research question, a large sample of 1,015 children was analyzed, although in two of the four approaches only a subset ( $n_{2}\;$ = 478) was analyzed because children with missing values could not be included when this statistical subtyping approach was used.

Limitations of previous studies (e.g., Salvador et al., 2019) were that only small sample sizes were analyzed and/ or only a few (mathematical) subdomains of performance were taken into account for subtyping. In contrast, in this research a large sample was analyzed and the analyses included a variety of mathematical skills as well as other cognitive areas: intelligence, working memory, and reading fluency. Furthermore, to check the results for robustness, four different subtyping approaches were conducted which differed with respect to which variables were included (all variables/only mathematical skills) and to what extent the measured results were aggregated into constructs (construct level) or considered as individual subtest results (subtest level).

On subtest level, three subgroups (by taking all variables into account) or six subgroups (by taking only mathematical variables into account) were identified. On construct level, two subgroups were found (by taking only mathematical variables into account). The results of these three analyses have in common that always one group was identified that showed severe and significant deficits in different mathematical skills: groups 2a, 3b and 2c. These three groups showed similar curves that reflect their cognitive profiles, and they consisted of disproportional many children who can be labeled as dyscalculic. This suggests that many children who are severely affected by arithmetic difficulties can be reliably distinguished from those without such difficulties – but some children identified as dyscalculic with the HRT 1–4 also appeared in other groups.

However, even though many children in the groups 2a, 3b, and 2c were dyscalculic, there was a considerable number of children in these subgroups that could not be identified as dyscalculic with the HRT 1–4 (Haffner et al., 2005) – regardless of whether the cut-off (PR) was set at 16, 10, or 5. This means, the boundaries between CwD and non-dyscalculic children appear to be fluid rather than strict. Nonetheless, dyscalculic and non-dyscalculic children tended to show different cognitive profiles. The assumption that the boundaries are fluid is also underlined by the fact that no subgroups were found in one analysis at construct level and thus children with and without dyscalculia could not be differentiated, here.

The cognitive profile of subgroup 3b showed some noticeable similarities to the cognitive profile that Kißler et al. (2021) found in their research project and that was called *subtype 2* by them: it was described as a severely impaired subtype in children with dyscalculia (Kißler et al., 2021). In this study, the cognitive profile of subgroup 2b (a group with mild deficits in some mathematical domains but without pronounced deficits in the non-mathematical domains) resembles the dyscalculia subtype that Kißler et al. (2021) named *subtype 1*: a subtype that showed minor deficits in comparison to the other subtype (*subtype 2*). This suggests that there is a group of children who are severely and unambiguously (presumably also persistently or long-term) impaired in their mathematical skills (subgroup 3b/subtype 2), while other children (subgroup 2b/subtype 1) show minor (and perhaps temporary) deficits in performing mathematical tasks. These two groups of children seem to differ.

With a view to these findings, it would be desirable if the definition of dyscalculia and the diagnostic criteria of this disorder were to be further developed in such a way that dyscalculia could not only be diagnosed on the basis of behavior or performance, but especially on the basis of more manifest criteria (e.g., specific neuronal divergences). This would make sense, as dyscalculia is classified as a neurodevelopmental disorder according to both the ICD-11 (World Health Organization, 2020) and DSM-5-TR (American Psychiatric Association, 2022). Therefore, a reliable way is needed to differentiate children with dyscalculia (a serious and long-term or persistent neurodevelopmental disorder as categorized in the ICD11: World Health Organization, 2020) from (1) other children with (temporary) deficits in mathematics/arithmetic that are of a different nature (e.g., temporary performance weakness due to challenging life circumstances) and (2) children in the normal range of development. Children who have an altered neuronal structure probably need different support than children who perform poorly in math for other reasons.

It can also be observed that children who scored very low on the HRT 1–4 (PR < 10 or PR < 5) achieved T-scores in the CODY-M 2–4 that correspond to a higher PR (Supplementary Table A1). However, this is not surprising considering the phenomenon of *regression to the mean*: This phenomenon occurs (especially with tests that are not perfectly correlated with each other, for example because they focus to varying degrees on different mathematical subdomains) when initially very high or very low scores are obtained and a subsequent measurement is taken (Barnett et al., 2005). This, in turn, leads to inconsistencies in the categorization of children as dyscalculic or non-dyscalculic, which once again highlights the need for more precise testing procedures.

Indeed, CwD seem to show structural divergences in special brain regions (e.g., in the parietal lobe, respectively, bilateral intra-parietal sulci) and these neurological divergences are hypothesized to be accountable for the core deficits in CwD (Butterworth, 1999; Dehaene et al., 2003; Landerl et al., 2004; Rykhlevskaia et al., 2009; Szűcs and Goswami, 2013). Nevertheless, there may also be approaches to foster mathematical competencies that are helpful for children with such neurological conditions as well as for children who have problems with acquiring arithmetic competencies for other reasons. This needs to be investigated in more detail.

On a first sight some results of this study may seem to contradict the results of Kißler et al. (2021) because the subgroups that were found in this study and which encompass many children with dyscalculia (group 2a, 3b, and 2c) do not only show deficits in terms of their mathematical competencies, but also below average results in reading fluency, intelligence, and working memory (i.e., matrix span). Kißler et al. (2021) were unable to find robust significant differences in these areas between the two subtypes they had characterized. But this does not have to mean that the severe impaired *subtype 2* was unimpaired in these areas, because the study by Kißler et al. (2021) lacks a comparison with unimpaired/ non-dyscalculic children. However, the mean *T*-scores of *subtype 2* were below the *T*-score of 50 in working memory, intelligence, and reading fluency (Kißler et al., 2021). This indicates below average performance in these areas. However, it should be noted that the deficits in reading fluency, intelligence, and working memory that were found in subgroups 2a, 3b, and 2c should not be understood as impairments in the narrower sense, but rather as weaknesses in comparison to the other subgroups, because, in summary, the *T*-scores are reduced but still tend to be in the lower normal range. Thus, when referring to a deficit (especially with a view to subgroup 2b), a reduced score relative to the other identified subgroups is meant.

In this study, the deficits that were shown by groups 2a, 3b, and 2c in reading fluency, intelligence, and working memory (matrix span) were significant in most analyses when frequentist statistics were used to compare these groups with other groups. These results were reconfirmed by Bayesian analyses. Therefore, these deficits of the groups 2a, 3b, and 2c appear robust and, in light of the findings by Kißler et al. (2021), consistent as well. Interestingly, group 2b (the group with less severe deficits in mathematical areas compared to group 3b) did not show those significant differences in working memory (matrix span), reading fluency and intelligence if compared to group 1b, group 4b, group 5b, and group 6b. Furthermore, group 2b showed significant better performance in these three performance areas than group 3b. These differences between group 2b and group 3b were reconfirmed by Bayesian analyses. In summary, these findings also suggest that there are substantial differences between children with more severe arithmetic difficulties (dyscalculia in the narrower sense) and children that tend to show lower performance in arithmetic. However, this lower performance subtype that was identified in the six-group solution (group 2b) was not identified in other approaches. Maybe, in the other analysis on subtest level the sample size was too small to detect this subtype.

Using model-based clustering-analyses to identify subgroups in children, Pieters et al. (2015) found different subgroups of children that differed qualitatively in terms of their arithmetic difficulties. Bartelet et al. (2014) also found different types of difficulties in mathematical skills that were characteristic for specific subtypes (e.g., the *weak mental number line subtype*, the *weak ANS subtype* and the *access deficit subtype*) in children with math learning difficulties (PR < 16 in an arithmetic fluency test). More detailed analyses on qualitative differences in arithmetic difficulties and/ or deficits in mathematical precursor skills as well as the severity of those could shed light on what distinguishes these subgroups. As already suggested in the introduction and in accordance with the results of Bartelet et al. (2014), different difficulties might be attributable to different underlying causes (e.g., deficits in the ANS vs. the access deficit hypothesis).

In contrast to Bartelet et al. (2014), no dyscalculia subtype or any other subgroup was found that was only or especially noticeable due to deficits in working memory. However, this difference could also be due to the fact that Bartelet et al. (2014) analyzed different working memory areas, whereas in this study only the matrix span was considered in the analyses.

It is striking that at construct level the results were different from the results at subtest level: In one analysis at construct level, it was not even possible to distinguish meaningful subgroups in any way because the one subgroup solution was the best solution. This is probably due to the fact that subgroups in the analyses at the subtest level showed the greatest differences in their transcoding skills. Thus, in particular this subtest served to differentiate the subgroups from one another. However, when the constructs were calculated, the variable *transcoding* merged into the variable for the construct *complex number processing*, which presumably involved a loss of information. Therefore, the information at the construct level was probably no longer sufficient to identify subgroups. This means that even the category *dyscalculia* could not be found exploratively as a separable group with this approach.

The three-subgroup solution (if subtyping was carried out by taking all variables into account on subtest level) and two-subgroup solution (if subtyping was carried out by taking only mathematical variables into account on construct level) have in common that each identified a single group with pronounced arithmetic difficulties but no other group with severe difficulties in mathematics. In the three-subgroup-solution, two-subgroup-solution, and six-subgroup-solutions, the group with the most severe arithmetic difficulties (2a, 3b, and 2c) showed better performance in BNP-tasks compared to their performance in calculation or tasks reflecting CNP. This suggests that dyscalculic children show severe deficits in arithmetic/calculating as well as in CNP but show less deficits in BNP tasks as magnitude comparison.

This findings for BNP have to be discussed. Children who were severely affected by arithmetic difficulties (groups 2a, 3b, and 2c) seem to have performed better in symbolic magnitude comparison and mixed magnitude comparison tasks – even achieved above-average results (groups 3b and 2c). This may initially seem peculiar, but in this study, an efficiency measure (median of correct response times/ number of correct responses) was used for these tasks, meaning that the goal was to respond both quickly and correctly. Careless errors could thus significantly impair the score. It can be assumed that children who are aware of their substantial difficulties in arithmetic (e.g., because of bad marks at school) try to compensate for their weaknesses by exerting extra effort and concentration/ attention, approaching these simple tasks more deliberately than children without such difficulties. Consequently, children with arithmetic difficulties might have made fewer errors, resulting in average or above-average performance – even if they then require a bit more time to solve the tasks. This could explain the observed phenomenon.

Why transcoding performance varies substantially across identified subgroups, and why it even seems to enable subgrouping, is an open question. The triple code model (Dehaene, 1992) suggests that transcoding/ transforming information from *auditory verbal word frame* (e.g., “thirteen”) into the *visual Arabic number form* (e.g., “13”) is something else than transforming an *analog magnitude representation* (e.g., “13 objects that are seen”) into the *visual Arabic number form*. Possibly, performance in transcoding verbal information into the visual Arabic number form could be a process that characterizes different subgroups of children at the children’s age examined in this study and possibly also allows predictions about their future development. This should be investigated in more detail by future studies.

The findings of this research highlight that children with dyscalculia appear to be heterogeneous. Therefore, dyscalculia does not seem to be a disorder with (1) a homogeneous cognitive profile and (2) a clear borderline to normality, making it difficult to systematically subgroup children with dyscalculia – much like children without dyscalculia are very heterogeneous in terms of their cognitive profiles, too.

## Data Availability

The raw data supporting the conclusions of this article will be made available by the authors, without undue reservation.
