# Supplementary material for: Identifying and subtyping dyscalculia in a sample of children with and without dyscalculia — a data-driven approach
Source: Front Psychol. 2025 Sep 23;16:1590581. doi: 10.3389/fpsyg.2025.1590581 (PMC12503033; doi:10.3389/fpsyg.2025.1590581)
Supplement: Supplementary file 1 [file Data_Sheet_1.pdf]

Christian Kißler, M. A., M. A. [1*], Prof. Dr. Jörg-Tobias Kuhn[1]

[1]Methods of educational research, Faculty of rehabilitation sciences, TU Dortmund, Dortmund, Germany

***Correspondence:**

Christian Kißler, M. A., M. A.

christian.kissler@tu-dortmund.de

(+49) 231/755 4554

TU Dortmund

44227 Dortmund

Emil-Figge-Str. 50

Germany

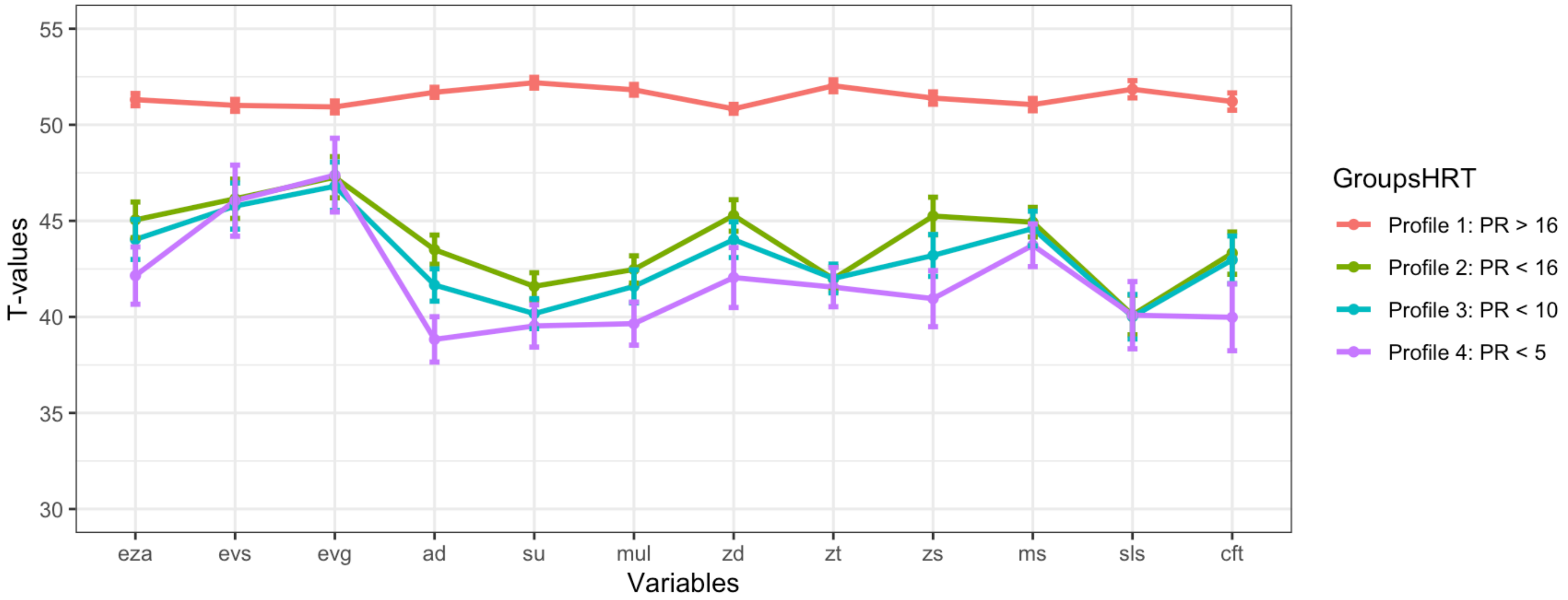

Figure A1: Visualization of descriptive statistics, subdivided by HRT 1-4 scores (percentile rank = PR)

Abbreviations: eza= dot enumeration; evs= symbolic magnitude comparison; evg= mixed magnitude comparison; ad= addition; su= subtraction; mul= multiplication; zd= transcoding; zt= number sets; zs= number line; ms= matrix span; sls= reading fluency; cft= intelligence; note: the means and standard errors are shown.

Table A1: Descriptive statistics, subdivided by HRT 1-4 scores (percentile rank = PR)

	HRT 1-4: PR > 16 (Group size: 870)			HRT 1-4: PR < 16 (Group size: 123)			HRT 1-4: PR < 10 (Group size: 93)			HRT 1-4: PR < 5 (Group size: 40)		
	M	SD	SE	M	SD	SE	M	SD	SE	M	SD	SE
Dot enumeration	51.31	9.38	0.32	45.05	10.21	0.93	44.03	10.04	1.04	42.15	9.45	1.49
Symbolic magnitude comparison	51.01	9.15	0.31	46.15	11.33	1.02	45.77	11.53	1.20	46.05	11.69	1.85
Mixed magnitude comparison	50.93	9.16	0.31	47.27	11.83	1.07	46.80	12.15	1.26	47.38	12.12	1.92
Addition	51.69	7.62	0.26	43.50	8.48	0.76	41.66	8.13	0.84	38.83	7.47	1.18
Subtraction	52.19	8.23	0.28	41.59	7.83	0.71	40.17	7.50	0.78	39.53	6.93	1.10
Multiplication	51.82	8.40	0.28	42.47	7.90	0.71	41.59	8.30	0.86	39.65	7.06	1.12
Transcoding	50.83	6.73	0.23	45.28	9.13	0.82	44.02	8.97	0.93	42.05	9.85	1.56
Number sets	52.03	9.29	0.31	41.98	7.08	0.64	42.00	7.28	0.75	41.55	6.45	1.02
Number line	51.39	9.45	0.32	45.25	10.85	0.98	43.20	10.55	1.09	40.95	9.20	1.46
Matrix span	51.05	9.55	0.32	44.93	8.55	0.77	44.60	8.71	0.90	43.73	7.03	1.11
Reading fluency	51.85	9.01	0.45	40.10	8.96	1.03	40.01	9.04	1.16	40.09	9.11	1.75
Intelligence	51.21	9.54	0.47	43.32	9.64	1.10	42.97	9.80	1.24	39.98	9.03	1.74

Table A2: Distribution of the children with dyscalculia across subgroups (clustered at subtest level, all variables were used for clustering)

	**Group 1a**	**Group 2a**	**Group 3a**
Group size	301 (2 children without test results in the HRT 1-4)	57 (1 child without test results in the HRT 1-4)	120
HRT < PR 16 ($\chi^2 = 87.30$, $p < 0.001$, Cramer's V = 0.40)	29 (= 9.70%)	33 (= 58.93%)	14 (= 11.67%)
HRT < PR 10 ($\chi^2 = 103.73$, $p < 0.001$, Cramer's V = 0.47)	18 (= 6.02%)	31 (= 55.36%)	12 (= 10.00%)
HRT < PR 5 ($\chi^2 = 54.02$, $p < 0.001$, Cramer's V = 0.34)	11 (= 3.68%)	15 (= 26.79%)	1 (= 0.83%)

Table A3: Distribution of the children with dyscalculia across subgroups (clustered at subtest level, mathematical variables were used for clustering)

	**Group 1b**	**Group 2b**	**Group 3b**	**Group 4b**	**Group 5b**	**Group 6b**
Group size	468 (13 children without test results in the HRT 1-4)	138 (1 child without test results in the HRT 1-4)	86 (3 children without test results in the HRT 1-4)	82 (1 child without test results in the HRT 1-4)	198 (2 children without test results in the HRT 1-4)	43 (2 children without test results in the HRT 1-4)
HRT < PR 16 ($\chi^2 = 79.78$, $p < 0.001$, Cramer's V = 0.28)	40 (= 8.79%)	25 (= 18.25%)	33 (= 39.76%)	13 (= 16.05%)	10 (= 5.10%)	2 (= 4.88%)
HRT < PR 10 ($\chi^2 = 95.06$, $p < 0.001$, Cramer's V = 0.31)	27 (= 5.93%)	20 (= 14.60%)	30 (= 36.14%)	10 (= 12.35%)	4 (= 2.04%)	2 (= 4.88%)
HRT < PR 5 ($\chi^2 = 80.09$, $p < 0.001$, Cramer's V = 0.28)	12 (= 2.64%)	8 (= 5.84%)	18 (= 21.69%)	0 (= 0.00%)	2 (= 1.02%)	0 (= 0.00%)

Table A4: Distribution of the children with dyscalculia across subgroups (clustered at construct level, mathematical variables were used for clustering)

	**Group 1c**	**Group 2c**
Group size	922 (19 children without test results in the HRT 1-4)	93 (3 children without test results in the HRT 1-4)
HRT < PR 16 ($\chi^2 = 78.18$, $p < 0.001$, Cramer's V = 0.28)	85 (= 9.41%)	38 (= 42.22%)
HRT < PR 10 ($\chi^2 = 90.48$, $p < 0.001$, Cramer's V = 0.30)	59 (= 6.53%)	34 (= 37.78%)
HRT < PR 5 ($\chi^2 = 69.93$, $p < 0.001$, Cramer's V = 0.27)	21 (= 2.33%)	19 (= 21.11%)
